# InAs Quantum Dots for Infrared Optoelectronics: Interface Engineering, Device Performance, and Emerging Opportunities

**DOI:** 10.3390/nano16211376

**Published:** 2026-10-27

**Authors:** Ling-Yi Ding, Wan-Shan Shen, Feng Zhao, Ya-Kun Wang, Liang-Sheng Liao

**Affiliations:** 1 Macao Institute of Materials Science and Engineering, Macau University of Science and Technology, Taipa, Macau SAR 999078, China; 2 Institute of Functional Nano & Soft Materials (FUNSOM), Jiangsu Key Laboratory for Carbon-Based Functional Materials & Devices, State Key Laboratory of Bioinspired Interfacial Materials Science, Soochow University, Suzhou 215123, China

**Keywords:** InAs quantum dots, interface engineering, device performance, infrared photodetectors, quantum dot light-emitting diodes, solar cells, field-effect transistors

## Abstract

Indium arsenide (InAs) quantum dots (QDs) offer a promising Pb- and Hg-free material platform for infrared optoelectronics owing to their narrow bulk bandgap, strong quantum confinement, and size-tunable optical response. However, surface defects, limited electronic coupling in quantum dot (QD) solids, and operational instability can prevent advances in material quality from translating into improved device performance. This review examines InAs QDs through a materials-interface-device-performance framework. We first discuss how synthetic control, core/shell heterostructures, ligand exchange, and surface reconstruction influence interfacial electronic states and carrier dynamics. We then analyze device-specific performance and trade-offs in solar cells, quantum dot light-emitting diodes, infrared photodetectors, and field-effect transistors, focusing on charge injection and extraction, trap-assisted losses, noise, and stability. Finally, we identify emerging opportunities in contact engineering, reliable performance benchmarking, scalable film fabrication, and advanced infrared device architectures. This device-oriented perspective emphasizes the role of coordinated interface engineering in connecting nanocrystal properties with functional optoelectronic performance.

## 1. Introduction

Infrared optoelectronic technologies play an increasingly important role in optical communication, biomedical imaging, environmental monitoring, and infrared sensing. Various semiconductor platforms have been developed to meet different spectral and device requirements, including conventional III-V semiconductors, type-II superlattices (T2SLs), and colloidal QDs. Epitaxial III-V materials, such as InSb and InAsSb, offer excellent carrier transport properties and established infrared device architectures, while InAs/GaSb and InAs/InAsSb T2SLs enable flexible band-structure engineering for infrared detection. However, their reliance on sophisticated epitaxial growth and precise interface control can increase fabrication complexity and limit compatibility with low-cost, large-area, and flexible electronics.

In contrast, colloidal QDs offer size-tunable optical properties and low-temperature solution processing, providing an alternative route toward scalable infrared optoelectronics. PbS and PbSe QDs have demonstrated promising near-infrared (NIR) to short-wave infrared (SWIR) performance, while HgTe QDs extend spectral coverage toward the mid-wave and long-wave infrared regions. Nevertheless, environmental and regulatory concerns associated with Pb- and Hg-containing materials have motivated the development of alternative QD systems. Among these, InAs QDs represent a promising Pb- and Hg-free semiconductor platform combining infrared spectral tunability with solution processability. Rather than universally replacing established infrared technologies, InAs QDs offer complementary opportunities for applications requiring low-temperature fabrication and potential compatibility with large-area processing or flexible substrates. However, their practical implementation remains constrained by surface defects, limited carrier transport in QD solids, and challenges associated with long-term device stability.

The distinctive potential of InAs QDs originates from their narrow bulk bandgap and large exciton Bohr radius, which enable strong size-dependent quantum confinement and spectral tunability from the NIR to the SWIR region. Depending on the QD size, composition, and surface/interface engineering strategies, colloidal InAs QDs have demonstrated tunable optical responses spanning approximately from 750 nm to beyond 1600 nm, with reported absorption extending toward 1700 nm in large-size QDs. The accessible spectral range is strongly influenced by synthetic control, size distribution, and carrier confinement effects, and the longest-wavelength operation typically requires larger QDs and more sophisticated surface passivation to mitigate defect-related losses. Furthermore, their solution processability enables low-temperature thin-film deposition and offers potential compatibility with large-area fabrication and flexible substrates. Benefiting from these characteristics, InAs QDs have been investigated in various optoelectronic devices, including quantum dot light-emitting diodes (QLEDs), infrared photodetectors, photovoltaic cells, and field-effect transistors. These advances demonstrate the versatility of InAs QDs for both light-emitting and light-detecting applications, highlighting their potential for future infrared optoelectronic systems.

As Pb/Hg-free III-V semiconductor nanocrystals, InAs QDs represent a promising alternative to conventional Pb/Hg-containing infrared QD materials. However, the environmental advantages of InAs-based devices depend on the complete device composition, including shell materials, dopants, processing chemicals, and end-of-life considerations. Core-only colloidal InAs QDs often suffer from surface-defect-mediated nonradiative recombination and oxidation sensitivity, whereas high photoluminescence quantum yield (PLQY) and improved stability generally require optimized synthesis, inorganic shell growth, and effective surface passivation. A key distinction should be drawn between a Pb/Hg-free, RoHS-oriented InAs core and a fully RoHS-compliant device stack: several high-performance structures discussed herein contain CdSe shells or Cd doping, and replacing Pb/Hg does not by itself resolve concerns related to arsenic-precursor toxicity, synthesis hazards, and end-of-life management.

**Figure 1:**
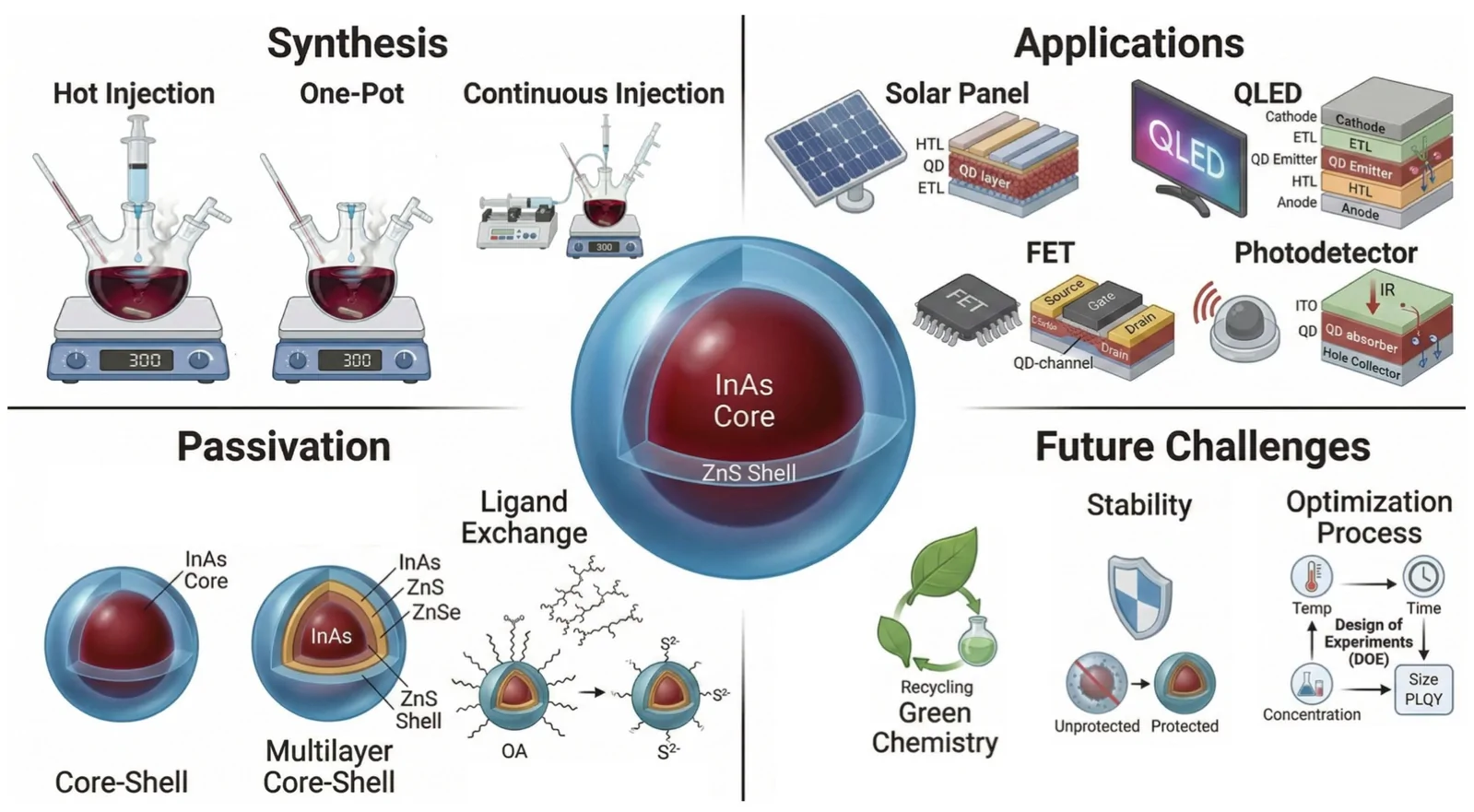
An overview of synthesis methods, passivation strategies, applications and challenges of InAs QDs.

Despite these advantages, the practical performance of InAs QDs remains strongly limited by their surface chemistry and interfacial properties. The large surface-to-volume ratio and incompletely coordinated surface atoms give rise to defect states that facilitate carrier trapping and nonradiative recombination, thereby reducing photoluminescence efficiency and compromising device performance. Moreover, their susceptibility to surface oxidation further complicates the realization of stable optoelectronic devices. To address these challenges, substantial efforts have focused on controlled synthesis, core–shell heterostructure construction, ligand engineering, and surface passivation to improve optical properties and charge transport. Nevertheless, improvements in one property do not necessarily translate into enhanced device performance. For example, insulating shells or long organic ligands can effectively suppress surface-related nonradiative recombination but may simultaneously hinder charge injection and transport. In addition, interfacial energy-level alignment, carrier selectivity, and defect formation at QD-QD and QD-transport-layer interfaces critically influence device operation. Therefore, understanding and engineering interfaces at multiple levels, from individual nanocrystals to complete device architectures, is essential for achieving an effective balance between optical efficiency, charge transport, and operational stability. Figure 1 provides an overview of InAs QD synthesis, passivation strategies, optoelectronic applications, and remaining challenges.

Recent reviews by Hu et al. [1] and Lin et al. [2] have comprehensively summarized advances in InAs QD synthesis, surface engineering, and optoelectronic applications. However, a device-oriented analysis is needed to clarify how different interfaces govern the dominant performance-limiting mechanisms in individual device architectures and to identify the trade-offs between optical performance and charge transport associated with different interface-engineering strategies. In this review, we adopt a device-driven, multilevel interface-engineering framework to critically analyze the relationships among synthetic control, interfacial chemistry, carrier dynamics, and optoelectronic functionality. Rather than providing another chronological summary of synthetic techniques or device-performance milestones, we focus on identifying the physical roles of different interfaces, the dominant loss mechanisms they introduce or suppress, and the trade-offs that emerge under different operating conditions. We further connect these mechanisms to device-performance metrics and identify emerging opportunities for scalable processing and application-specific architectures. System-level integration is addressed only through directly reported examples rather than assumed to be established for InAs QD technologies as a whole.

## 2. Materials and Interface Engineering of InAs QDs

The optoelectronic performance of InAs QD-based devices is closely connected to the chemical history of the nanocrystals. Precursor reactivity, reaction temperature, and growth kinetics influence the evolution of nanocrystal size, crystallinity, and surface composition. Variations in surface stoichiometry, particularly the distribution of undercoordinated In and As sites, can modify ligand-binding behavior and the susceptibility of QD surfaces to oxidation. These effects influence the formation of electronic trap states and the degree of electronic doping or Fermi-level pinning in QD solids.

Surface engineering provides a means of modifying these chemical and electronic characteristics. Appropriate ligand coordination and inorganic passivation can reduce electronically active defects and suppress nonradiative recombination. Meanwhile, ligand exchange can alter interparticle spacing, electronic coupling, film conductivity, and interfacial dipoles. However, these effects are not necessarily mutually reinforcing. For example, strongly bound or electronically insulating ligands may improve surface passivation while limiting interparticle charge transport, whereas aggressive ligand exchange can enhance electronic coupling but introduce new defects or destabilize the surface.

These material-dependent properties further influence carrier transport and recombination at device interfaces. In photodetectors, surface defects and interfacial energy barriers can contribute to carrier trapping, leakage currents, and noise, thereby affecting responsivity, response speed, and detectivity. In QLEDs, trap-assisted recombination, unbalanced charge injection, and interfacial quenching can reduce radiative efficiency and operational stability. In photovoltaic devices, unfavorable band alignment and interfacial recombination can limit charge separation and collection, leading to losses in open-circuit voltage and power conversion efficiency (PCE).

**Figure 2:**
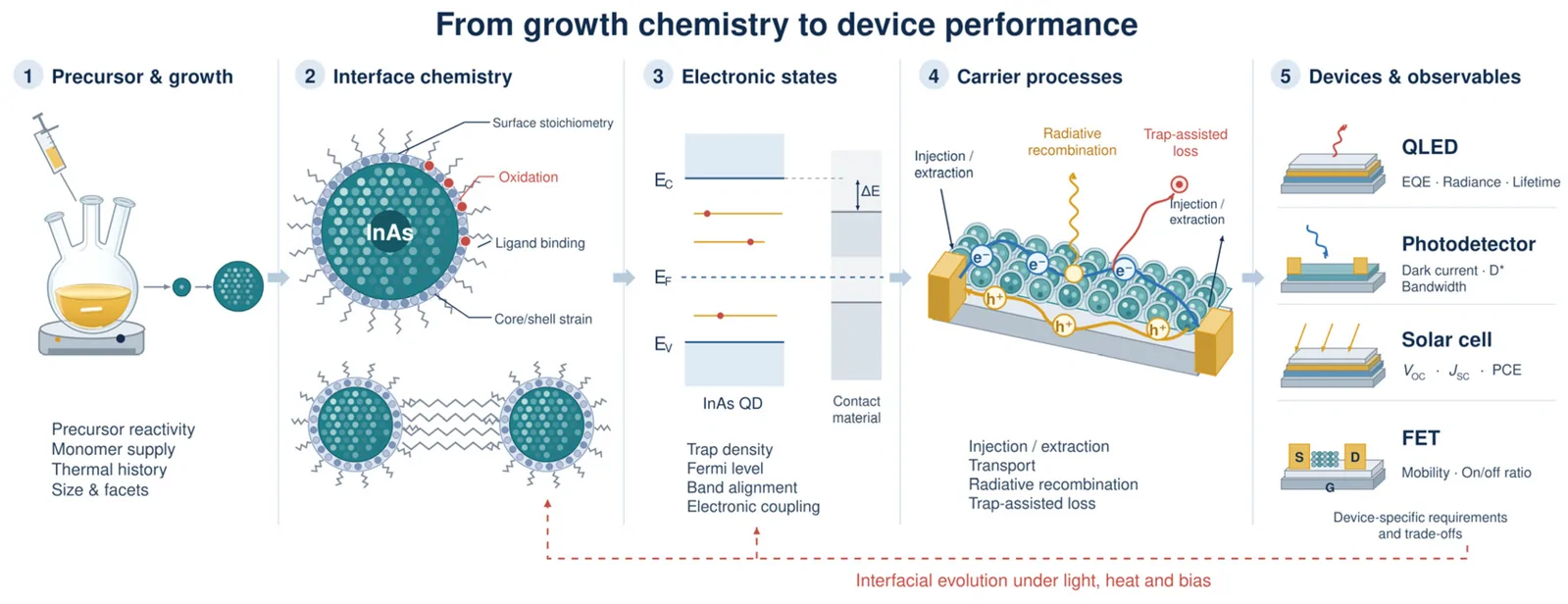
Unified materials-interface-device-performance framework for InAs QD-based infrared optoelectronics.

Therefore, the optimization of InAs QD devices requires coordinated control over nanocrystal synthesis, surface stoichiometry, ligand chemistry, and interfacial energetics. Rather than optimizing individual processing steps in isolation, a materials-interface-device approach offers a more effective basis for understanding performance variations across different studies and identifying transferable strategies for device improvement. Figure 2 schematically illustrates this unified materials–interface–device–performance framework, highlighting how synthetic control and interface engineering collectively govern carrier dynamics and device performance in InAs QD-based infrared optoelectronics.

### 2.1. Synthetic Control and Device-Relevant Surface Chemistry

Synthetic control determines the size, crystallinity, and initial surface chemistry of InAs QDs, thereby setting the conditions for subsequent shell growth, ligand exchange, and film formation. The central synthetic challenge is to match precursor conversion with nucleation and growth while avoiding a broad size distribution and defective surfaces. Hot-injection and one-pot approaches address this challenge through precursor selection, reducing-agent chemistry, and temperature control [3,4,5,6,7,8,9]. For example, less reactive arsenic precursors can moderate monomer generation, whereas aminoarsine-based routes require appropriate reductive activation. These choices should be evaluated through their effects on both optical uniformity and the chemical state of the resulting surface, rather than through the synthesis method alone.

Feed-controlled growth provides a complementary means of separating nucleation from subsequent size evolution. Continuous precursor injection regulates monomer supply, while preformed InAs clusters serve as reservoirs that release growth species under suitable reaction conditions [10,11,12,13,14,15,16]. Matching the supply rate to nanocrystal growth can maintain size focusing and suppress secondary nucleation, enabling larger QDs with extended infrared absorption. Amine coordination and diffusion control further modify this growth window [11,17,18]. From an interface-engineering perspective, these approaches provide control over the nanocrystal population on which later surface treatments act; narrow absorption features alone, however, do not establish that the QDs will form a low-defect, electrically connected solid.

Alternative precursor and reduction routes broaden the available processing options and can influence surface quality during growth. Representative approaches include in situ arsenic-source generation, zinc-assisted co-reduction, and adjustment of the indium-to-arsenic precursor ratio [19,20,21]. Zinc coordination has been used to modify InAs surface properties and improve photodiode performance [20], while carboxyl-free synthesis offers a route to suppressing oxide formation at its origin [22]. The significance of these advances for devices is that synthesis can reduce the burden on subsequent defect-removal treatments. Their suitability still depends on reproducible surface composition, compatibility with purification and ligand exchange, and the requirements of the intended device.

The relationship between synthesis and the final interface is illustrated by Ban et al. [18]. InAs QDs prepared using different arsenic precursors displayed different amounts of surface-bound indium oleate, despite comparable target sizes. Tailored InBr3-based resurfacing treatments brought their surface stoichiometries and photodetector performance into closer agreement. This result indicates that a surface inherited from synthesis is a starting condition that can be modified by postsynthetic chemistry. Consequently, precursor selection and surface reconstruction should be considered together when establishing a reproducible route from colloidal QDs to functional thin films.

For device-oriented materials selection, the relevant outcome is therefore a QD population whose spectral response meets the application requirements and whose surfaces tolerate the subsequent processing needed for effective passivation and charge transfer. Table 1 summarizes representative synthetic methods for InAs QDs, highlighting the effects of precursor selection and reaction conditions on QD size and optical properties, including excitonic absorption and emission ranges, spectral linewidths, peak-to-valley (P/V) ratios, and PLQY. The following subsections focus on how inorganic shells, surface ligands, and device contacts convert these synthetic starting conditions into controlled carrier confinement, recombination, and transport.

### 2.2. Core/Shell and Strain-Managed Heterostructures

The luminescent properties of InAs QDs are commonly evaluated in terms of PLQY, emission full width at half maximum (FWHM), and optical stability. PLQY represents the ratio of emitted to absorbed photons under optical excitation. Some studies use the term photoluminescence quantum efficiency (PLQE) to describe the same quantity. In this review, PLQY is adopted as the preferred terminology, while PLQE is retained when referring to the original terminology or figure labels in the cited literature. In addition to the numerous non-radiative surface defect states introduced by dangling bonds on the surface of bare core QDs, the optically active luminescent core surface is also susceptible to interference from external water, oxygen, and thermal energy. When QDs are continuously exposed to oxygen molecules under light excitation for extended periods, it induces the formation of surface defect states, leading to permanent fluorescence quenching of the QDs. QD fluorescence can be quenched when heated in an inert gas atmosphere and mostly recover to its original state upon cooling; however, when exposed to air, thermal energy causes irreversible degradation of the QDs. Therefore, while improving the fluorescence QY and FWHM performance of QDs, their stability issues cannot be ignored. Core/shell engineering has emerged as an effective strategy for enhancing the luminescence efficiency and stability of InAs QDs by suppressing surface-related nonradiative recombination and protecting the emissive core from its surrounding environment.

**Figure 3:**
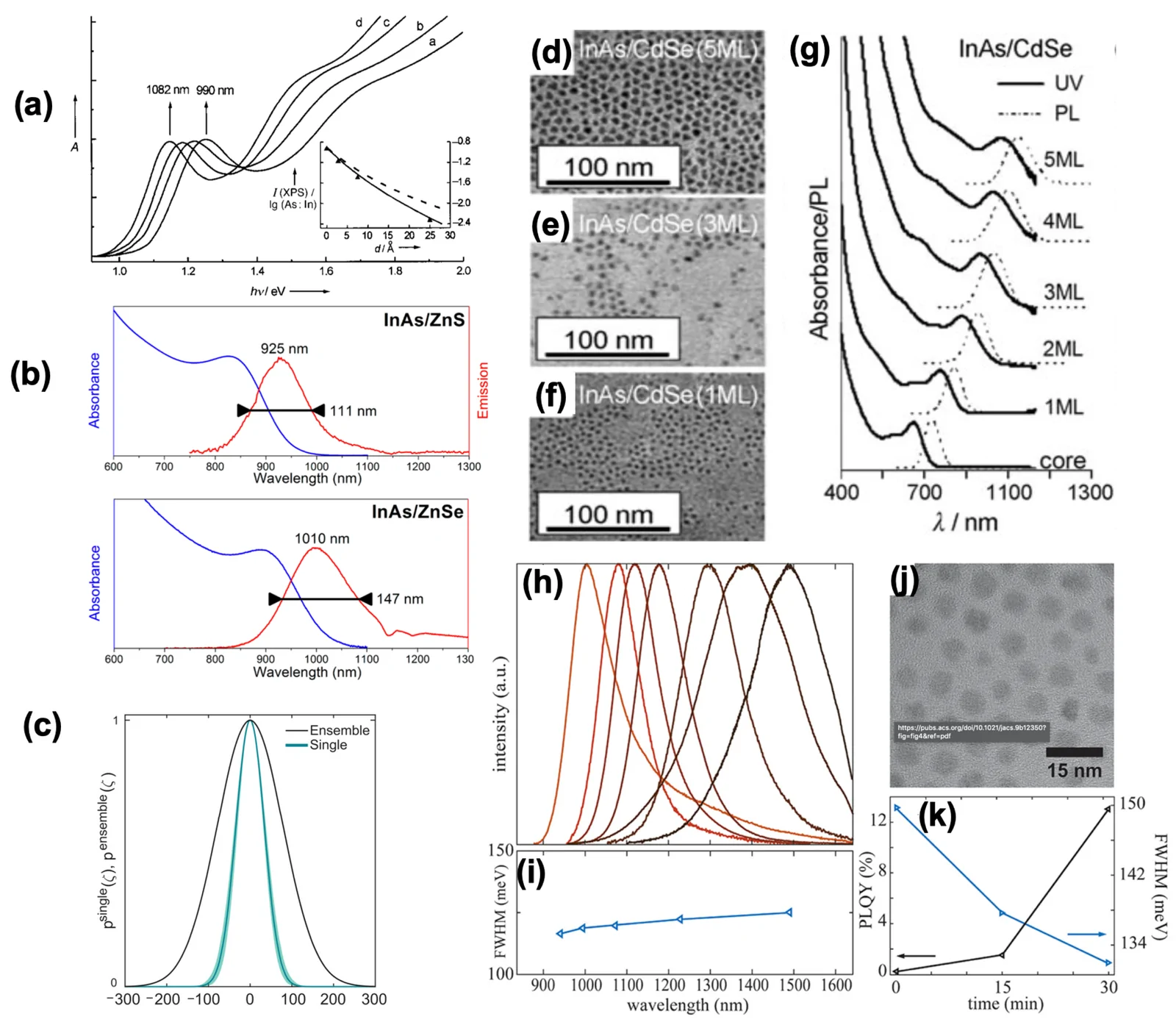
(**a**) Absorption spectra of InAs core and InAs core/shell nanocrystals during shell growth. The arrows indicate characteristic absorption features at approximately 1082 and 990 nm during the shell growth on InAs QDs. [25]; reprinted with permission from [25]. Copyright 1999, WILEY-VCH Verlag GmbH, Weinheim, Fed. Rep. of Germany. (**b**) Absorption and emission spectra of InAs/ZnS and InAs/ZnSe QDs. The horizontal arrows indicate the FWHM of the emission spectra, corresponding to 111 nm for InAs/ZnS QDs and 147 nm for InAs/ZnSe QDs. [6]; reprinted with permission from [6]. Copyright 2016, American Chemical Society. (**c**) Gaussian fits of the single InAs/CdSe NC (blue) and ensemble (black) spectral correlations [27]; reprinted with permission from [27]. Copyright 2019, American Chemical Society. (**d**–**f**) TEM images of InAs/CdSe QDs; (**g**) UV/Vis absorption and PL spectra of QDs with shells of different thicknesses; reprinted with permission from [26]. Copyright 2008, WILEY-VCH Verlag GmbH & Co. KGaA, Weinheim. (**h**) Size-dependent tunability of the emission properties of InAs/CdSe core–shell QDs; (**i**) The PL FWHM remains narrow at around 120 meV throughout the whole tunability range; (**j**) HR-TEM characterization of core–shell particles; (**k**) typical progression of PLQY and PL FWHM during shell growth procedure [7]; reprinted with permission from [7]. Copyright 2020, American Chemical Society.

Core-only InAs QDs are particularly susceptible to surface-related defects because of their large surface-to-volume ratio and undercoordinated surface atoms. Inorganic shell passivation can substantially reduce the density of electronically active surface defects, thereby suppressing nonradiative recombination and improving PLQY and optical stability. Additionally, the different bandgap widths of the core and shell materials in core–shell QDs result in varying carrier confinement capabilities, leading to different spatial distributions of carrier wave functions in core–shell QDs. Based on the characteristics of electron-hole spatial distribution, core–shell QDs can be classified into three types: Type-I, Type-II, and quasi-Type II. In Type-I QDs both electrons and holes are located in the core, with highly localized wave functions and high electron-hole spatial overlap. InAs/CdSe is the most typical Type-I core–shell QD [25]. They constructed InAs/InP and InAs/CdSe core–shell structures through TOP solution passivation, finding that the small conduction band (CB) offset of CdSe and InP shells led to electron wave function extension into the shell, causing a redshift in the absorption spectrum (Figure 3a). However, the fluorescence QY performance of the two core–shell structures differed significantly. The InP shell reduced QY by 8 times, while the CdSe shell increased QY by 18 times (the absolute QY up to 20%). Xie and Peng demonstrated that epitaxial CdSe shell growth through a one-pot approach substantially improved the optical performance of InAs QDs, increasing the PLQY from below 1% for the initial QDs to approximately 90% after shell formation. This result highlights that the high luminescence efficiency originates from effective shell passivation rather than being an intrinsic characteristic of the core-only InAs QDs [26]. As the shell grew, the size of the nanocrystals increased (Figure 3d–f), and the UV/Vis and PL spectra of the resulting core–shell nanocrystals exhibited a substantial red shift (Figure 3g). The resulting passivated InAs/CdSe core/shell QDs exhibited improved stability in air and retained their luminescence in aqueous environments after thiol ligand exchange under the reported experimental conditions, providing an ideal Pb/Hg-free NIR luminescent material for biomedical imaging and optical communication applications. Although CdSe shells significantly improve optical properties and stability, these structures should not be considered fully heavy-metal-free systems because cadmium-containing components introduce additional environmental and regulatory concerns.

Bertram et al. found that increasing the thickness of the InAs/CdSe shell led to a decrease in single-particle fluorescence linewidth to 52 meV (Figure 3c) [27]. Ginterseder et al. prepared InAs/CdSe core–shell structures with central emission wavelengths between 1000 and 1500 nm (Figure 3h) and PL FWHM of about 120 meV (Figure 3i) [7]. Figure 3j shows HR-TEM images of the core–shell particles while Figure 3k illustrates the typical evolution of PLQY and FWHM during shell growth for a sample whose final emission peaks at 1250 nm. Compared to core-only particles, the synthesized core–shell particles consistently show better uniformity in both shape and size. InAs/ZnSe and InAs/ZnS are also common Type-I core–shell QDs. ZnSe and ZnS have larger band offsets that enhance carrier confinement, improving the oxidation resistance and photostability of the core–shell structure. Zhang and Zhang reported that introducing zinc stearate together with the indium source not only enhanced the photoluminescent properties of InAs QDs without causing doping, but also promoted their one-pot growth into InAs-based core/shell structures [19]. The PLQY of InAs/ZnS and InAs/ZnSe core/shell QDs was 8 and 15%, respectively. In 2016, Grigel et al. proposed a passivation method for InAs/ZnS and InAs/ZnSe core–shell structures: using Zn(stearate)2 and TOP-S/TOP-Se as precursors, they grew the shell layer via a one-pot method, enabling QDs to emit in the NIR region with fluorescence QY of 5–10% [6]. As shown in Figure 3b, InAs/ZnS QDs exhibited an absorption feature at approximately 925 nm and a PL emission peak at 1111 nm, whereas InAs/ZnSe QDs showed red-shifted absorption and emission features at approximately 1010 and 1147 nm, respectively. Zhu et al. demonstrated that ZnCl2 can act as a Z-type ligand to passivate In vacancies and induce ZnSe shell formation in situ, spontaneously constructing an In-Zn-Se interface layer to alleviate mismatch and reduce surface traps, raising the PLQY of Cd-free InAs/ZnSe to ≈42% (≈860 nm) [28].

Thicker shells mean more precursor materials in the reaction system, which can lead to anisotropic growth of QDs, resulting in spectral broadening. Therefore, further design of core–shell structures is needed to reduce structural stress at the core–shell interface, slow down the energy increase required for strained shell lattice thickness, and prevent the process from exceeding the energy required to form mismatch defects. To mitigate lattice mismatch-induced strain (e.g., 7% between InAs and ZnSe), multi-shell systems were developed.

**Figure 4:**
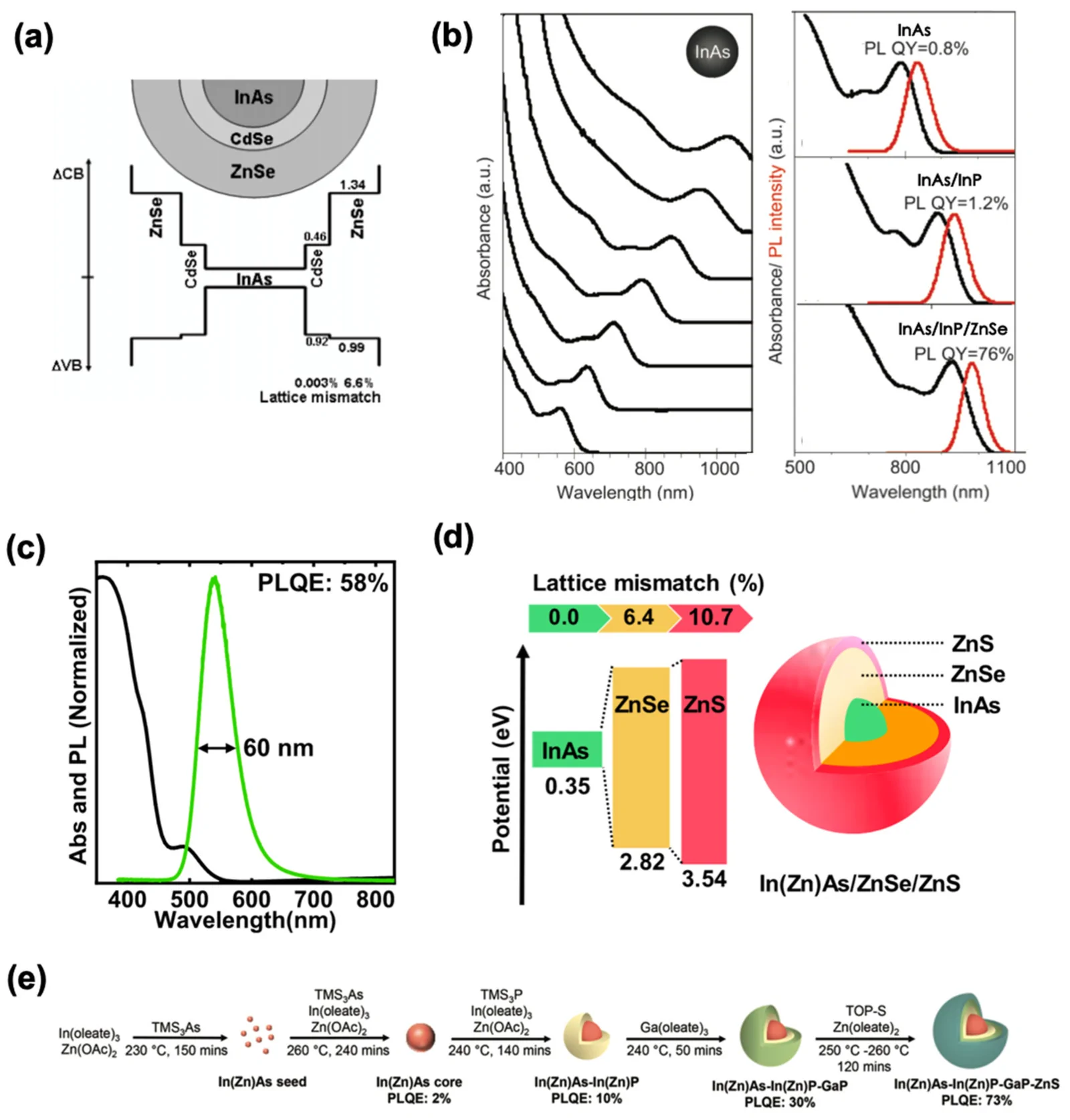
(**a**) Diagram of the potential structure for the InAs/CdSe/ZnSe core/shell/shell structure [29]; reprinted with permission from [29]. Copyright 2006, American Chemical Society. (**b**) UV–vis-NIR absorption spectra of InAs QDs with different sizes and the absorption/PL spectra measured upon the successive addition of the InAs core–shell QD [12]; reprinted with permission from [12]. Copyright 2008, Tsinghua Press and Springer-Verlag GmbH. (**c**) Absorption and photoluminescence spectra of In(Zn)As/ZnSe/ZnS QDs, showing a PLQE of 58%; (**d**) band alignment and lattice diagram of the In(Zn)As/ZnSe/ZnS core–shell QD [32]; reprinted with permission from [32]. Copyright 2021, American Chemical Society. (**e**) Synthesis and structural and spectroscopic characterization of In(Zn)As/In(Zn)P/GaP/ZnS QDs [33]. Reprinted with permission from [33]. Copyright 2022, Wiley-VCH GmbH.

The early multilayer shell structure passivation strategy was proposed by Aharoni et al., who used a cadmium-containing InAs/CdSe/ZnSe structure (Figure 4a) [29]. This structure effectively alleviated the up to 7% lattice mismatch between the InAs core and the ZnSe shell through a CdSe buffer layer, while the outer ZnSe provided a CB offset of up to 1.3 eV, strongly confining carriers. This strategy was remarkably effective, significantly increasing the QY of small-sized InAs cores from 4 to 70%, and, even after mercaptopropionic acid (MPA) ligand exchange for water solubility, it maintained a QY of 37% and good photostability.

To address the environmental and regulatory concerns associated with cadmium-containing shells, Pb/Hg-free passivation schemes have become an important research focus. A typical example is the InAs/InP/ZnSe structure reported by Xie et al., which uses InP as a lattice buffer layer and ZnSe as a protective shell [12]. This structure can achieve tunable emission from 700 to 1100 nm, with PLQY as high as 40–90% (Figure 4b). Enright et al. conducted an in-depth study and optimization of this system, stabilizing its PLQY at 60 ± 2%, but they also pointed out that carrier occupation in the InP shell and unpassivated phosphorus dangling bonds at the interface are key challenges limiting further QY improvement [30].

Another Pb/Hg-free structure, InAs/ZnSe/ZnS, was extensively studied by Zhang et al. [31]. They found that although the double shell could increase QY to 25%, the extremely small effective mass of electrons in InAs allowed the electron wave function to easily extend to the surface, making the QDs highly sensitive to surface oxidation and resulting in poor luminescence stability. Darwan et al. synthesized visible-light-emitting In(Zn)As/ZnSe/ZnS QDs using a low-temperature nanocluster method, achieving a high QY of 58% (Figure 4c). Figure 4d shows the band alignment and lattice diagram of the In(Zn)As/ZnSe/ZnS core–shell QD, demonstrating the potential of this structure for applications in different spectral bands [32].

To further improve performance and stability, more complex quaternary “giant shell” structures have been developed. Wijaya et al. reported InAs/In(Zn)P/ZnSe/ZnS and In(Zn)As/In(Zn)P/GaP/ZnS structures [34]. Their innovation lies in introducing GaP as a lattice buffer layer, significantly suppressing lattice mismatch and interface defects between InP and the outermost ZnS. This design was supported by research from Sagar et al., who found that introducing a GaP intermediate layer could increase PLQY by about 1.7 times and achieve extremely narrow emission linewidths (about 100 meV) [35]. Zhao et al. used similar In(Zn)As-In(Zn)P-GaP-ZnS core–shell QDs (Figure 4e) to increase PLQE to 73% and successfully fabricated efficient SWIR-LEDs with an external quantum efficiency (EQE) of 13.3% [33]. In 2025, high-performance NIR QD-LEDs were achieved by Li et al. using high-quality, RoHS-oriented InAs-based QDs. These QDs were incorporated into an electroluminescent device that featured tailored hole-transport layers (HTLs). By employing zinc fluoride as a critical additive, large and monodisperse InAs/InP/ZnSe/ZnS core/multi-shell QDs were synthesized, achieving a nearly 100% PLQY. The resulting QD-LEDs delivered a peak EQE of 20.5% at 905 nm, a radiance of 581.4 W·sr^−1^·m^−2^, and a half-lifetime (T_50_) of 550 h at 50 W·sr^−1^·m^−2^ [36].

From a device-performance perspective, the function of an inorganic shell extends beyond the suppression of surface defects and the enhancement of PLQY. Core/shell interfaces simultaneously regulate band offsets, carrier-wavefunction distributions, lattice strain, and the accessibility of charge carriers to surrounding materials. These factors may impose competing requirements on the design of optoelectronic devices. For light-emitting applications, sufficiently effective carrier confinement and the elimination of nonradiative recombination centers are generally desirable because they favor exciton formation and radiative decay within the emissive region. Multi-shell architectures with appropriately engineered interfaces can therefore provide substantial improvements in emission efficiency and environmental stability. However, the presence of an additional wide-bandgap shell may also affect carrier injection and interparticle electronic coupling, depending on shell composition, thickness, energy-level alignment, and device configuration. For photodetection, photovoltaic, and transistor applications, efficient charge transport and carrier extraction can be equally important. Consequently, maximizing optical confinement does not necessarily provide the optimal interface for electronic transport. The most suitable core/shell structure must therefore balance defect passivation against the carrier-transfer requirements of the intended device.

These considerations illustrate that shell composition, strain management, and thickness should be evaluated not only through optical indicators such as PLQY and spectral linewidth, but also through their consequences for carrier injection, extraction, transport, and operational stability. Establishing such structure-interface-function relationships is essential for transferring high-quality InAs QDs from colloidal dispersions into electrically functional architectures.

### 2.3. Ligand Exchange and Surface Reconstruction

In addition to inorganic shell growth, ligand exchange and surface reconstruction provide direct control over the chemical environment of InAs QDs. These strategies regulate surface stoichiometry, passivate undercoordinated atoms, and improve interparticle electronic coupling. Surface passivation and ligand exchange strategies for InAs QDs are crucial for their application in bioimaging and optoelectronic devices. Over the years, researchers have developed a series of methods to enhance their stability, luminescence efficiency, and charge transport performance.

Early representative work was completed by Allen et al., who developed a polymer imidazole ligand exchange strategy [37]. The team used a random copolymer containing polyethylene glycol and imidazole groups to replace the original ligands, successfully achieving water solubility for InAs/ZnCdS core–shell QDs which provide improved optical properties but contain cadmium components. This method maintained a QY of about 25% while controlling the hydrodynamic diameter to below 10 nanometers, significantly improving biocompatibility. The QDs were stable in serum and had reduced cytotoxicity, with an in vivo blood circulation half-life of up to 110 min, providing an excellent probe platform for live molecular imaging.

**Figure 5:**
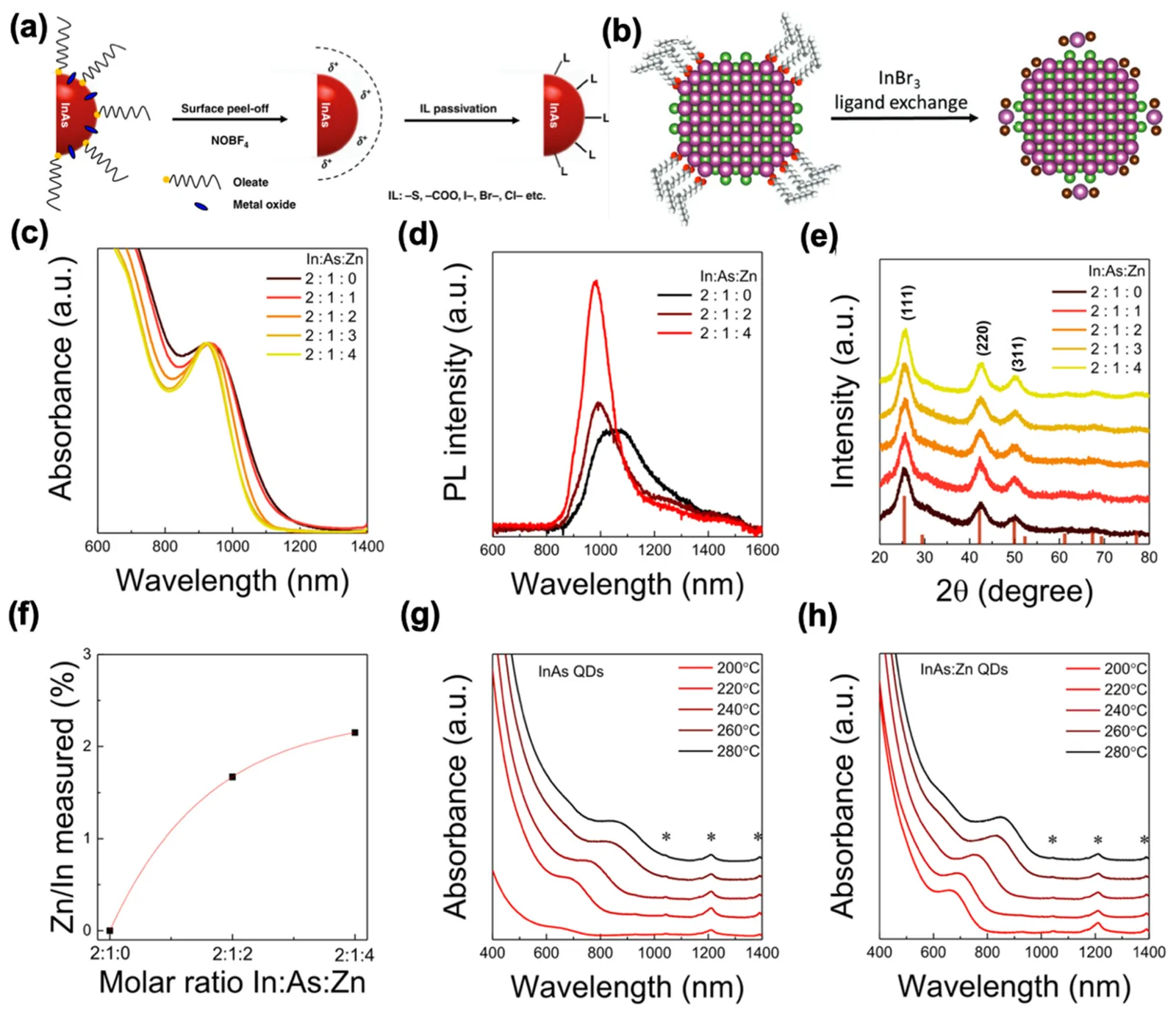
(**a**) Schematic illustration of the two-step surface modification process for InAs QDs [17]; reprinted with permission from [17]. Copyright 2018, The Authors. (**b**) InBr_3_ ligand exchange scheme and InAs QD surface structure [38]; reprinted with permission from [38]. Copyright 2022, Wiley-VCH GmbH. (**c**–**h**) Impact of Zn precursor type on InAs QD optical and structural properties (* indicate the excitonic absorption features) [20]; reprinted with permission from [20]. Copyright 2023, The Authors.

To further enhance the optoelectronic performance of QDs and their potential for device applications, Song et al. proposed a two-step surface modification method [17]. They first used nitrosyl tetrafluoroborate (NOBF_4_) to thoroughly remove oleic acid ligands and the oxide layer from the QD surface, completing “surface cleaning”. Then, in N,N-dimethylformamide solvent, they used halide ions (Br^−^/Cl^−^/I^−^) or short-chain thiols (such as 3-mercaptopropionic acid and 1,2-ethanedithiol) for ligand reconstruction (Figure 5a). This treatment not only significantly restored PL intensity but also confirmed successful ligand binding through ζ-potential measurements, laying the foundation for subsequent efficient device fabrication.

Addressing the challenge of poor charge transport performance in QD solid films, Sun et al. introduced an innovative amphoteric passivation strategy [38]. They replaced traditional insulating ligands with InBr_3_ and added N,N-dimethylformamide as a coordination stabilizer (Figure 5b). InBr_3_ dissociates into Br^−^ (X-type ligand) to passivate indium dangling bonds and InX_2_^+^ (Z-type ligand) to passivate arsenic dangling bonds, achieving dual-site synergistic passivation of the InAs surface. With improved surface passivation, this method boosted the mobility of InBr_3_-InAs QD solids to 10 times that of halide-exchanged InAs solids.

Given the specific energy requirements for III-V QD surface passivation, Koh et al. explored vapor phase treatment as a new approach. Building on NOBF_4_ solution treatment, they further treated QD films with H_2_S gas at 150–250 °C to form a sulfide layer on the surface [39]. This high-temperature vapor phase treatment effectively overcame the limitations of solution treatment, providing new insights for deeper surface passivation and more efficient charge transport.

The latest advancement comes from Kim et al., who took a novel approach by directly introducing zinc coordination complexes during the synthesis of InAs QDs [20]. Figure 5c–h show the impact of Zn precursor type on InAs QD optical and structural properties. Zinc ions indirectly participated in the reaction and ultimately passivated the QD surface, which not only improved colloidal stability but also significantly enhanced their electrical performance. Infrared photodiodes made from these Zn-passivated InAs QDs showed a dark current reduced by two orders of magnitude compared to unpassivated samples, achieving efficient detection of 1050 nm NIR and SWIR.

An important distinction in evaluating ligand-exchange strategies is the difference between chemical surface passivation and the formation of electronically functional QD solids. The former primarily concerns the removal or compensation of undercoordinated surface sites and oxidation-related defects, whereas the latter additionally requires sufficiently strong interparticle coupling, controlled energetic disorder, and compatible interfaces with adjacent device layers.

For example, replacing long-chain organic ligands with shorter inorganic or molecular species can reduce the separation between neighboring QDs and facilitate carrier transport. At the same time, ligand binding can modify surface dipoles, band-edge positions, and charge-carrier concentrations. Such changes may improve carrier injection or extraction in one device architecture while producing unfavorable band alignment or leakage pathways in another. Therefore, ligand exchange should be understood as a combined process of surface reconstruction, electronic-structure modulation, and thin-film interface formation rather than simply as a means of increasing carrier mobility.

This distinction is particularly important when interpreting experimental measurements. Enhanced PLQY or prolonged photoluminescence lifetime provides evidence of reduced nonradiative losses under the corresponding optical measurement conditions, but does not independently establish improved carrier transport in a solid film. Conversely, improved field-effect mobility may indicate more favorable electrical transport without necessarily demonstrating suppressed interfacial recombination in an operating photodiode or LED.

A more comprehensive evaluation of surface-engineering strategies should therefore combine chemical and structural analysis with optical, electrical, and device-level measurements. Relevant approaches include X-ray photoelectron spectroscopy for surface composition, grazing-incidence scattering for film organization, time-resolved spectroscopy for carrier relaxation, and electrical characterization for trap-related transport and charge extraction. Correlating these complementary measurements provides a more reliable basis for identifying the interfacial mechanisms responsible for device improvements.

Although surface passivation and core/shell engineering have substantially improved the stability of InAs QDs, their long-term operation remains limited by several degradation pathways, including photo-oxidation, thermal degradation, and electrically induced aging. Photo-oxidation is one of the primary challenges for colloidal InAs QDs because surface-exposed In and As atoms can undergo oxidation under light irradiation and oxygen-containing environments. The formation of surface oxides introduces additional trap states within the bandgap, which promote nonradiative recombination and accelerate photoluminescence quenching. In particular, the small effective mass of electrons in InAs facilitates electron wavefunction extension toward the surface, making insufficiently passivated QDs more vulnerable to oxidative degradation. Inorganic shell growth, improved surface stoichiometry, and robust ligand coordination can mitigate these processes by reducing surface defect density and limiting oxygen diffusion.

Thermal degradation represents another important limitation, especially during device fabrication and continuous operation. Elevated temperatures can induce ligand desorption, interfacial atomic diffusion, and structural reconstruction of QDs, leading to increased defect formation and deteriorated carrier confinement. In core/shell structures, thermal stress caused by lattice mismatch between the core and shell materials may further generate interfacial defects when the thermal budget exceeds the tolerance of the heterostructure. Therefore, strain management through compositionally graded shells, buffer layers, and optimized shell thickness is essential for maintaining structural integrity under thermal stress.

Electrical aging becomes increasingly significant when InAs QDs are incorporated into practical optoelectronic devices. Under continuous electrical bias, charge accumulation, trap filling, and ion migration at QD-ligand or QD-transport-layer interfaces can gradually modify energy-level alignment and increase leakage pathways. In light-emitting devices, charge imbalance may accelerate Auger recombination and interfacial degradation, whereas in photodetectors, persistent trapping and defect-mediated leakage can increase dark current and reduce detectivity. Consequently, improving operational stability requires not only effective QD surface passivation but also comprehensive interface engineering, including stable charge-transport layers, optimized energy alignment, and suppression of defect evolution during device operation.

Collectively, ligand exchange and surface reconstruction play essential roles in regulating the surface chemistry and electronic properties of InAs QDs. Polymer-based ligands primarily improve colloidal stability and biocompatibility, whereas halide and short-chain thiol treatments can enhance surface passivation and interparticle charge transport. Amphoteric passivation, vapor-phase treatments, and metal-ion coordination further provide effective strategies for suppressing surface defects and improving electronic properties. However, improved surface passivation does not necessarily lead to enhanced device performance, as changes in ligand chemistry can also affect electronic coupling and energy-level alignment. Therefore, optimizing surface engineering strategies requires balancing defect passivation, charge transport, and stability to meet the specific requirements of different optoelectronic devices.

### 2.4. Interparticle and Device-Contact Interface Engineering

Device-level interface engineering begins with the assembly of surface-engineered InAs QDs into continuous films and the formation of contacts with charge-transport layers or electrodes. The resulting inter-QD interfaces must provide sufficient electronic coupling while retaining the passivation established in solution. Amphoteric and sequential co-passivation strategies illustrate how coordination chemistry can support conductive InAs QD solids [36,40]. For a practical film, residual ligand content, packing, coverage, and thickness must also be controlled: local gaps can create leakage paths, whereas excessive thickness can increase the distance carriers must travel before collection. This makes film formation part of interface engineering and requires optical improvements in colloidal dispersions to be reassessed after deposition.

The next requirement is to establish carrier-selective contacts. Ligand-induced changes in surface dipoles and carrier concentration can shift the electronic alignment of an InAs QD film relative to neighboring layers, as demonstrated by the ligand-dependent junction properties of InAs-based photovoltaics [17]. A contact should facilitate the desired carrier transfer while limiting unwanted injection and interfacial recombination. These objectives differ among architectures: an LED requires balanced electron and hole injection into the emissive layer, whereas a photodiode requires selective extraction together with suppressed dark injection. In a transistor, source/drain injection and trapping at the channel/dielectric interface must be considered alongside transport between QDs. Thus, the same surface treatment cannot be assumed to produce an equally favorable contact in every device.

Processing compatibility places additional constraints on these interfaces. Sequential deposition requires solvent and treatment conditions that preserve the underlying layers, while thermal processing must remain compatible with the substrate and the assembled stack. For example, Li et al. used photo-crosslinked blended hole-transport materials to improve hole transfer and enable multilayer solution processing in InAs QD LEDs [36]. By contrast, the sulfide-vapor treatments reported by Koh et al. operate at 150–250 °C [39], so their use in a particular device stack requires an assessment of its thermal tolerance. For larger-area or patterned devices, an appropriate extension of this analysis is to evaluate film uniformity, solvent exposure, and retention of passivation throughout the complete fabrication sequence.

An imaging-compatible photodiode stack provides a further example of how interfacial defects and processing compatibility influence device performance. Siddik et al. reported a thin-film PIN heterojunction InAs colloidal quantum dot (CQD) photodiode stack incorporating amorphous indium gallium zinc oxide and copper iodide transport layers, with a spectral response extending to approximately 1200 nm [41]. The device exhibited a dark current density of 4.7 μA cm^−2^ at −1 V and 298 K, which decreased to 3.6 nA cm^−2^ at 220 K. Importantly, the authors identified deep electronic trap states and frequency-dependent trapping-detrapping noise, highlighting the need to control electronic defects when translating InAs CQD materials into integrated photodiode stacks. The study further demonstrated infrared imaging through monolithic integration of the photodiode with a silicon readout integrated circuit. This early demonstration supports the feasibility of coupling solution-processed InAs CQD photodiodes with silicon readout electronics, while the observed trap-related noise underlines the importance of interface control for reliable imaging performance.

The completed interface must also remain chemically stable during operation. Xia et al. identified zinc migration from a ZnO electron-transport layer (ETL) as a degradation pathway in InAs QD photodetectors and addressed it through changes to the transport-layer architecture [42]. This example shows why initial energy-level alignment and QD passivation are insufficient criteria for selecting adjacent materials. Device-performance evaluation should therefore connect surface and film characterization with measurements of contact losses, recombination, leakage, and stability under the intended operating conditions. These requirements provide the basis for the device-specific analysis in Section 3, where each architecture is examined through its operating requirements, critical interfaces, dominant loss mechanisms, engineering strategies, and performance trade-offs.

## 3. Device Performance and Applications of InAs QDs

### 3.1. Solar Cells

QD solar cells are considered an important candidate for third-generation photovoltaics due to their low-cost solution processing and the unique design flexibility offered by quantum confinement. Among the material systems, InAs QDs stand out in colloidal III-V semiconductors due to their small bandgap and large exciton Bohr radius. Their energy bands can be continuously tuned from the visible to the NIR and even SWIR range through size and surface chemistry engineering. This, along with their high absorption coefficient, controllable morphology, and large electric dipole moment, helps enhance light absorption and exciton separation, thereby expanding the spectral response window of solar cells.

**Figure 6:**
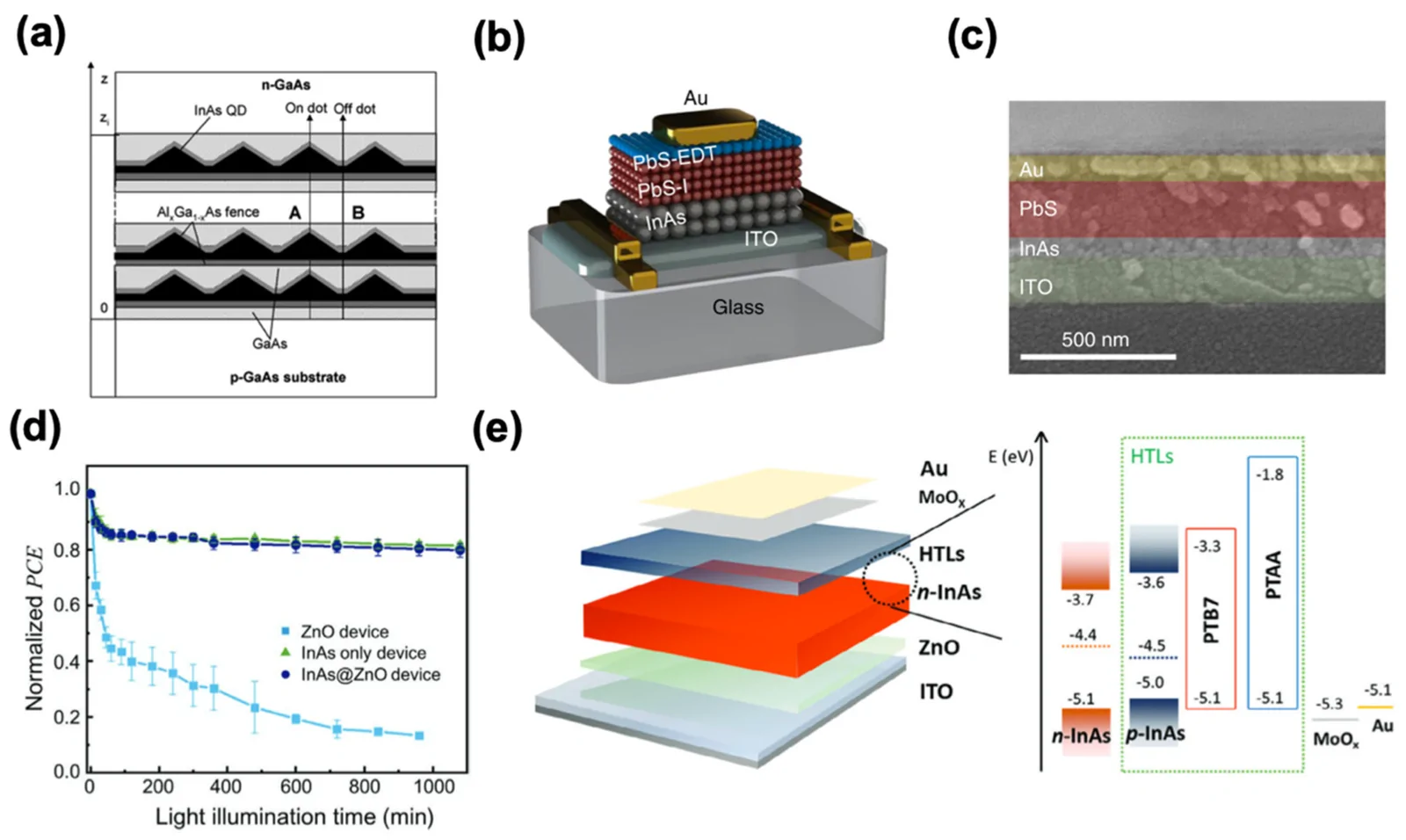
(**a**) QD arrangement in the fence barrier solar cell structure (Path A represents on-dot charge transport through the GaAs buffer, AlₓGa_1-x_As fences, InAs wetting layers, and QDs, whereas Path B represents off-dot transport through the same layers, excluding the QDs) [45]; reprinted with permission from [45]. Copyright 2007, American Chemical Society. (**b**) Schematic architecture of p-n junction photovoltaics using n-type InAs QD layers; (**c**) SEM image of photovoltaics [17]; reprinted with permission from [17]. Copyright 2018, The Authors. (**d**) Ambient-air stability test of five PM6:Y6 BHJ OPV devices under constant 1 sun illumination [51]; reprinted with permission from [51]. Copyright 2022 Wiley-VCH GmbH. (**e**) Schematic device structure of InAs homojunction photovoltaics and energy levels of HTLs in InAs photovoltaics structure [52]; reprinted with permission from [52]. Copyright 2024, Wiley-VCH GmbH.

In 1997, Luque and Martí theoretically investigated intermediate-band solar cells (IBSCs) using a detailed-balance model [43]. Under maximum optical concentration (approximately 46,050 suns), the theoretical conversion efficiency of an ideal IBSC can reach 63.2%, compared with 40.7% for an ideal conventional single-junction solar cell under the same conditions. This theoretical advantage arises from the additional absorption of sub-bandgap photons through an intermediate electronic band. InAs QDs can be embedded into GaAs single-junction cells as intermediate band materials. In 2004, the first intermediate band solar cell was successfully fabricated, consisting of InAs QDs sandwiched between ordinary N-type and P-type semiconductors [44]. Wei and Forrest calculated the intermediate band solar cells with InAs/GaAs QDs having an AlGaAs barrier structure (Figure 6a). The AlGaAs barrier made the InAs QDs a true center for photogenerated carrier generation and collection, rather than a recombination region [45]. In 2008, Martí et al. found that the open-circuit voltage of QD intermediate band solar cells was lower than that of ordinary single-junction solar cells without QDs [46]. They attributed this to the reduced recombination in the intermediate band, with recombination from the CB to the valence band (VB) becoming the main component. The intermediate band InAs QDs could not effectively absorb photons with energy below the bandgap to generate photocurrent. Additionally, the introduction of InAs QDs increased defects in the cell structure, leading to increased non-radiative recombination and thus reducing the open-circuit voltage. In the same year, Hubbard et al. improved conversion efficiency and reduced dark current by inserting GaP strain compensation layers between multiple layers of InAs QDs, achieving a slight increase in short-circuit current density compared to GaAs single-junction solar cells [47].

In 2011, Christopher G. Bailey et al. used GaP and GaAsP strain compensation techniques to achieve an open-circuit voltage close to 1V, with a 3.5% increase in short-circuit current compared to GaAs single-junction solar cells. In 2012, Tutu et al. from University College London found that using high-temperature spacer layers of GaAs could also improve the performance of QD solar cells [48]. In the same year, Gu et al. from the Institute of Semiconductors, Chinese Academy of Sciences, found through theoretical research that changing the position of the InAs QD layer in the i-layer could improve the efficiency of QD intermediate band solar cells. Besides serving as intermediate band materials, InAs QDs can also be inserted as i-type layers into Ga(In)As sub-cells to enhance the long-wave response of Ga(In)As sub-cells, improving current matching in conventional GaInP/Ga(In)As/Ge triple-junction solar cells and thus increasing their efficiency [49]. In 2014, Kerestes et al. incorporated 10 layers of InAs QDs into the (In)GaAs subcell of GaInP/(In)GaAs/Ge triple-junction solar cells, achieving an increase of 0.20 mA cm^−2^ in the integrated sub-bandgap photocurrent density, as determined from quantum efficiency measurements [50].

In 2018, Song et al. developed solar cell devices based on InAs QDs by constructing a p-n junction with n-type InAs QD layers and p-type lead sulfide QD layers (Figure 6b,c) [17]. This structure achieved efficient carrier extraction through band optimization, obtaining a certified energy conversion efficiency of 7.92% without additional charge transport layers. Specifically, researchers prepared defect-controlled conductive InAs QD films through a two-step surface modification process with halogen bromide ions and 3-mercaptopropionic acid (MPA). The current-voltage curve showed that solar cells without InAs QD layers had an energy conversion efficiency of less than 2% due to low built-in potential, while devices with bromide InAs-Br and InAs-MPA QDs achieved efficiencies of 4.61% and 7.23%, respectively. The efficiency improvement of InAs-MPA QD devices was attributed to enhanced built-in potential (energy level shift), increased carrier concentration, and improved charge transport performance. The EQE indicated that the efficiency improvement in the short-wave region was particularly significant for InAs-MPA QD cells compared to bromide InAs devices and devices without QD layers.

In 2022, Park et al. incorporated InAs QDs as an ETL in PM6 organic photovoltaic devices, achieving a PCE of 15.1% [51]. In this architecture, PM6 serves as the primary photoactive absorber, whereas InAs QDs function as a charge-selective interfacial layer that facilitates electron extraction and improves operational stability. Therefore, the reported efficiency represents the performance of an InAs-assisted organic solar cell rather than an InAs QD absorber-based photovoltaic device. As shown in Figure 6d, the cell maintained over 80% of its initial efficiency after 1000 min of one-sun illumination under ambient conditions. In contrast, control samples based on zinc oxide electron transport layers degraded to less than 20% of their initial efficiency under the same conditions, demonstrating that the InAs QD layer effectively suppressed photodegradation. Fourier transform infrared spectroscopy analysis of PM6:Y6 films on glass/zinc oxide and glass/InAs QD substrates revealed that films on InAs QD substrates maintained stable infrared characteristic peaks after illumination, while PM6 and Y6 donor materials on zinc oxide substrates decomposed due to superoxide radicals and high-energy photons. Further studies on the effect of different QD layer thicknesses showed that, when the InAs QD film thickness increased to 20 nanometers, the device’s fill factor and shunt resistance improved, reaching peak efficiency. However, further thickening reduced the light absorption capacity of the PM6:Y6 active layer due to excessive QD density, leading to a decrease in short-circuit current density. Devices with optimized thickness maintained over 85% of their initial performance after continuous illumination in air, demonstrating that narrow-bandgap InAs QDs with discrete energy levels can block high-energy photons and selectively collect electrons, thereby simultaneously enhancing efficiency and stability.

In 2025, Park et al. developed InAs CQD solar cells by combining multifunctional ligand passivation with n-p homojunction engineering [52]. Initially, n-type InAs CQD absorbers were treated with 3-mercaptopropionic acid (MPA) and ZnCl_2_, where MPA enhanced colloidal stability and ZnCl_2_ further passivated surface defects and reduced the n-type doping concentration. Compared with the single-ligand-passivated InAs (InAs-SL) devices, the multifunctional-ligand-passivated InAs (InAs-MLs) devices exhibited an increase in PCE from 0.85% to 1.80%, accompanied by improvements in open-circuit voltage (V_OC_), short-circuit current density (J_SC_), and fill factor (FF). Subsequently, a conductive p-type InAs CQD layer was introduced to form an n-p homojunction with the n-type InAs-MLs absorber (Figure 6e), further suppressing interfacial recombination and enhancing charge extraction. This homojunction architecture increased V_OC_ to 0.42 V and PCE to 2.40% for the large-bandgap InAs CQD solar cells. Similarly, the ZnCl_2_ passivation strategy was applied to p-type InAs QDs, achieving a peak EQE of 41% at 970 nm. Thickness optimization experiments showed that when the p-type layer thickness exceeded 20 nanometers, it effectively suppressed charge recombination between the n-type absorption layer and the electrode, achieving a high open-circuit voltage of over 0.3V. However, thicknesses exceeding 40 nanometers led to increased recombination within the QD layer, causing a decrease in fill factor. As the p-type layer thickened, the EQE peak redshifted due to the interference effect of reflected and transmitted waves caused by changes in the optical cavity length. Although the current efficiency of InAs QD cells is comparable to advanced QD cells, there is still room for improvement in stability. The photovoltaic performance of representative InAs QD-based solar cells and other QD photovoltaic devices is summarized in Table 2.

These advancements underscore the potential of InAs QDs in developing high-efficiency solar cells based on Pb-free InAs QD materials, ranging from intermediate-band photovoltaics to organic and homojunction devices and paving the way for their widespread application in next-generation photovoltaic technologies. Through strategic engineering of surface passivation, strain compensation, band alignment, device architecture, and synthesis methods, the photovoltaic performance of InAs QDs continues to improve, positioning them as a versatile key material in the field of solar energy harvesting.

### 3.2. Quantum Dot Light-Emitting Diodes (QLEDs)

InAs quantum dots have become an important research focus in the development of high-performance QLEDs, particularly for NIR and SWIR applications. Compared with conventional II-VI and IV-VI quantum dots, InAs-based emitters based on appropriately designed compositions can provide a pathway toward RoHS-compatible infrared emitters with size-tunable emission across 850–1600 nm, making them highly promising for next-generation infrared light sources in covert communication, wearable health monitoring, machine vision, and compact sensing. As discussed in Chapter 2, continuous advances in InAs QD synthesis, core/shell structural design, and surface passivation have directly promoted improvements in device performance. Improvements in PLQY, emission linewidth, and colloidal stability, achieved through optimized nanocrystal synthesis, core/shell engineering, and surface passivation, provide an important foundation for efficient electroluminescence.

**Figure 7:**
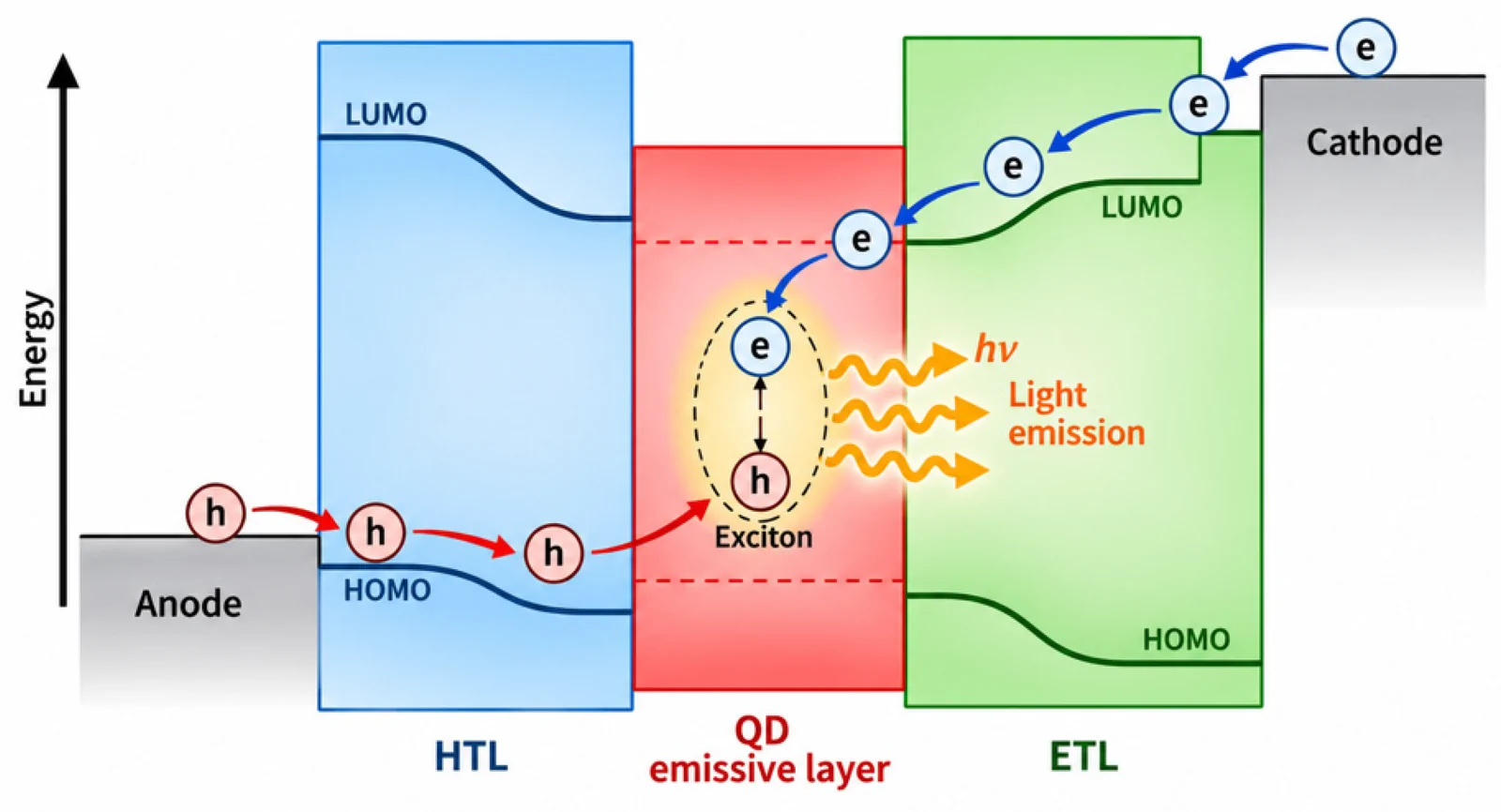
Luminescence mechanism of QLED devices.

As illustrated in Figure 7, the operating mechanism of QLEDs generally involves three consecutive processes: carrier injection, charge transport, and exciton recombination. Under forward bias, electrons are injected from the cathode into the ETL, while holes are injected from the anode through the HTL. Driven by the applied electric field, electrons and holes migrate toward the QD emissive layer, where they recombine within the nanocrystals to form confined excitons. Radiative recombination of these excitons generates photons. However, non-radiative processes, including surface trap-mediated quenching, Auger recombination induced by charge imbalance, and exciton energy transfer to adjacent charge transport layers, can significantly limit device efficiency. EQE is the ratio of the total number of photons emitted by the QLED device to the number of injected electrons, satisfying the following relationship [60]:

*EQE* = *γη_rad_η_out_*

Therefore, the *EQE* of QLEDs is mainly determined by the charge balance factor *γ*, the radiative recombination efficiency *η_rad_*, and the light outcoupling efficiency *η_out_*, among which *η_rad_* is closely related to the PLQY of the QDs. In addition to EQE, radiance, current efficiency, turn-on voltage, and operational half-lifetime T_50_ are also important metrics for evaluating QLED performance.

Although substantial progress has been achieved in InAs QD-based electroluminescent devices, extending the emission wavelength from the NIR to the SWIR region remains challenging in terms of both device efficiency and operational stability. Recent NIR InAs QLEDs have achieved EQEs exceeding 20%, whereas comparable efficiencies at longer SWIR wavelengths remain difficult to achieve. This performance difference cannot be simply attributed to increasing QD size but reflects the combined effects of nanocrystal growth, surface defect chemistry, nonradiative recombination, and charge injection dynamics.

Extending the emission wavelength generally requires larger InAs QD cores with reduced quantum confinement. However, maintaining a narrow size distribution during prolonged growth can become increasingly challenging because variations in precursor reactivity, growth kinetics, and surface reconstruction may broaden the particle size distribution. Such variations can introduce energetic disorder and broaden the emission spectrum, potentially promoting carrier localization in lower-energy QDs. Meanwhile, although the surface-to-volume ratio decreases with increasing core size, changes in surface facets, ligand coverage, and oxidation susceptibility can influence the distribution and electronic activity of surface defects. Consequently, the effective passivation of larger InAs QDs remains critical for maintaining high PLQY.

In addition to surface-related losses, the narrower energy gap associated with longer-wavelength emission can increase the importance of nonradiative recombination pathways. In particular, multiphonon-assisted relaxation and other energy-gap-dependent nonradiative processes may become more competitive with radiative recombination. Trap-assisted recombination further reduces the fraction of injected carriers contributing to photon emission. Moreover, Auger recombination can cause substantial efficiency losses under conditions of high carrier density or charge imbalance, particularly when QDs accumulate excess electrons or holes during electrical operation. These processes collectively limit the achievable PLQY and electroluminescence efficiency, although their relative contributions depend on the QD structure, surface passivation, and operating conditions.

At the device level, extending the emission wavelength can also modify the energy-level alignment between InAs QDs and adjacent charge-transport layers. Changes in QD band-edge positions, together with interfacial dipoles and ligand-induced energy shifts, can alter electron and hole injection barriers and lead to unbalanced carrier accumulation. Such imbalance may enhance nonradiative recombination and Auger losses, thereby reducing EQE. Furthermore, interfacial defects, charge accumulation, and electrically induced chemical changes can accelerate device degradation, making operational stability an additional challenge for longer-wavelength SWIR QLEDs.

Therefore, achieving efficient SWIR electroluminescence requires coordinated optimization of nanocrystal growth, surface and shell passivation, energy-level alignment, and device architecture. In particular, controlling core size dispersity, suppressing trap-assisted and Auger recombination, and balancing carrier injection are essential for improving EQE while preserving long-term operational stability. Representative device performance metrics across different emission wavelengths are summarized in Table 3.

The earliest InAs-based electroluminescence was reported by Tessler et al., who employed InAs/ZnSe core/shell QDs as the emissive layer to construct solution-processed polymer-nanocrystal hybrid LEDs with tunable emission in the range of 1.0–1.3 μm [63]. This pioneering work demonstrated the feasibility of printable NIR QLEDs based on InAs nanocrystals. Meanwhile, it also revealed an important early insight: the polymer matrix could suppress PLQY quenching between closely packed QDs. However, owing to insufficient carrier transport in the hybrid emissive film, the device EQE was limited to approximately 0.5%, with a relatively high turn-on voltage of about 15 V. This performance gap indicated that achieving high-performance InAs QLEDs requires simultaneous enhancement of QD emissive properties through shell and surface passivation, together with optimization of carrier transport and device architecture.

**Figure 8:**
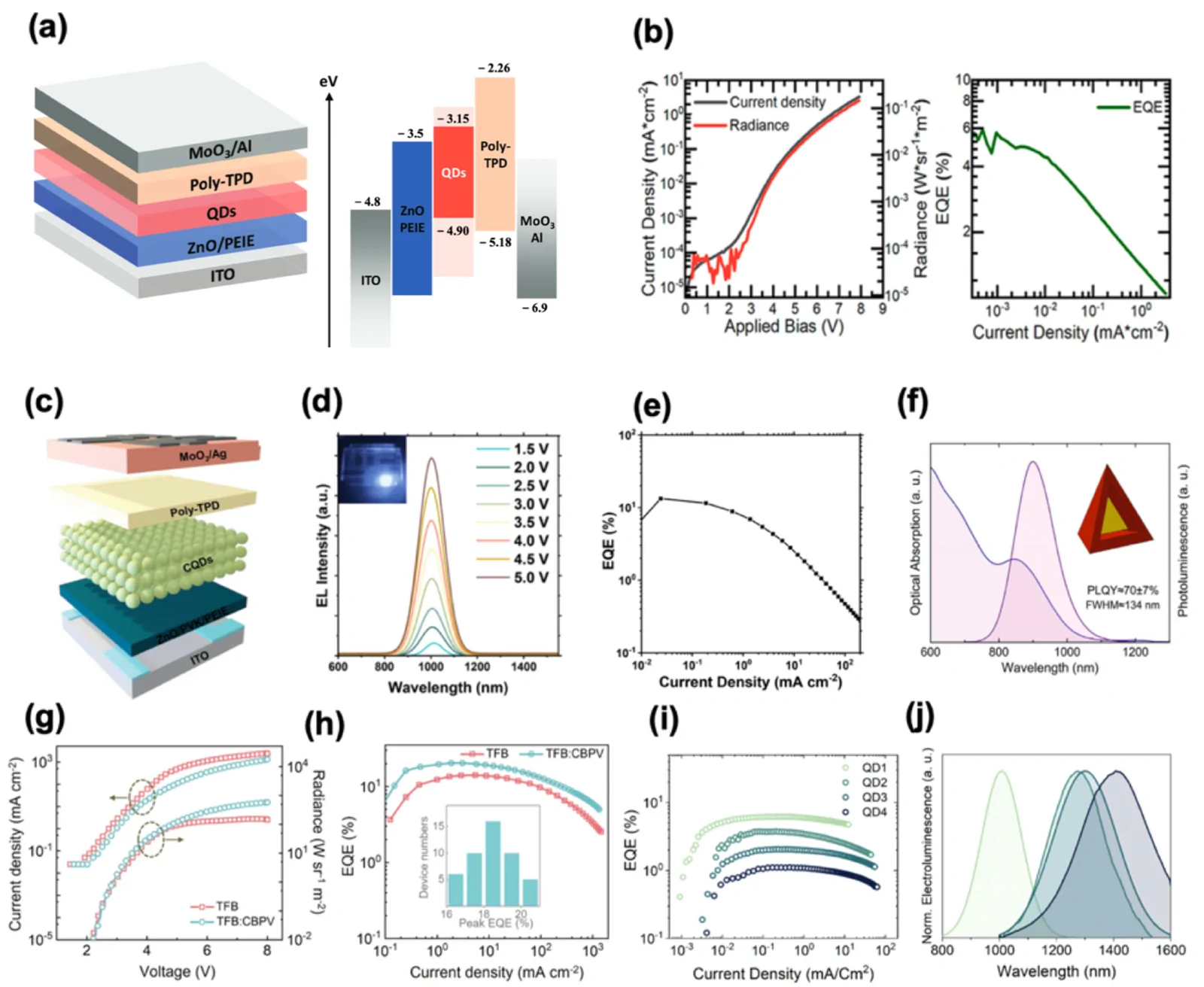
(**a**) QD-LED device structure ITO/ZnO/PEIE/QD/Poly-TPD/MoO_3_/Al and the associated energy band diagram [34]; reprinted with permission from [34]. Copyright 2019, WILEY-VCH Verlag GmbH & Co. KGaA, Weinheim. (**b**) Radiance (red solid curve) versus applied bias and EQE versus current density [23]; reprinted with permission from [23]. Copyright 2022, The Authors. (**c**) SWIR LED device structure ITO/ZnO/PVK/PEIE/QDs/Poly-TPD/MoO_3_/Ag; (**d**) EL spectra of LEDs recorded at different applied voltages. Inset: Photograph of an operating LED emitting in the SWIR region.; (**e**) EQE versus current density of the SWIR LED [33]; reprinted with permission from [33]. Copyright 2022, Wiley-VCH GmbH. (**f**) Emission and optical absorption characteristics of the InAs/ZnSe QDs [61]; reprinted with permission from [61]. Copyright 2024, The Authors. (**g**) Current density and radiance for NIR QD-LEDs based on TFB HTLs (light coral) and TFB:CBPV HTLs (sky blue); (**h**) EQE vs voltage for the corresponding NIR QD-LEDs [64]; reprinted with permission from [64]. Copyright 2025, The Authors. (**i**) EQE versus current density of the devices; (**j**) EL spectrum of QLEDs [62]; reprinted with permission from [62]. Copyright 2026, The Authors.

Over the following two decades, the performance of InAs QLEDs continued to improve, mainly benefiting from the combined progress in core/shell engineering and device stack design. In 2020, Wijaya et al. reported a Pb/Hg-free QLED based on In(Zn)As/In(Zn)P/GaP/ZnS core/multi-shell QDs with a device structure of ITO/ZnO/PEIE/QD/Poly-TPD/MoO_3_/Al (Figure 8a), achieving an EQE of 4.6% and a maximum radiance of 8.2 W·sr^−1^·m^−2^ at 850 nm [34]. This work demonstrated that rational device engineering, such as the introduction of an inorganic ZnO ETL, a conjugated polymer HTL, and interfacial modification layers, can effectively improve carrier injection and charge balance in Pb/Hg-free NIR QLEDs. Subsequently, with the development of safer aminoarsine precursor chemistry, De Franco et al. further synthesized RoHS-compliant InAs/ZnSe core/shell QDs via the aminoarsine route and fabricated devices with an EQE of 5.5% and a radiance of 0.15 W·sr^−^^1^·m^−^^2^ at 947 nm (Figure 8b) [23]. These results further demonstrated the feasibility of Pb- and Hg-free InAs QDs for NIR electroluminescent applications.

A highly efficient SWIR LED based on In(Zn)As/In(Zn)P/GaP/ZnS core/multi-shell colloidal QDs, reported by Zhao et al., represents an important advance in this field [33]. The key materials innovation of this work lies in the design of a multi-shell structure with gradually decreasing lattice constants, allowing lattice strain in the core/shell structure to be progressively released. In particular, the GaP interlayer acts as a lattice buffer between In(Zn)P and ZnS, effectively mitigating interfacial defect formation and facilitating the growth of a thick, high-quality ZnS outer shell. This core/multi-shell structure achieved a high PLQE of 73% at around 985 nm. In addition to materials design, this work also introduced an important device-level optimization by incorporating an unconventional PVK interlayer on the electron-injecting side, forming an ITO/ZnO/PVK/PEIE/QDs/Poly-TPD/MoO_3_/Ag device structure (Figure 8c). Although PVK is generally regarded as a hole-transporting semiconductor, when positioned between ZnO and the QD layer, it can electronically decouple the QDs from ZnO, suppress interfacial luminescence quenching, and block excessive electron injection, thereby improving the charge balance factor γ. As a result, the device achieved an EQE of 13.3% at 1006 nm (Figure 8d–e) and exhibited good operational stability, retaining 85% of its initial EQE after 20 h of continuous operation. This work established the importance of graded core/shell lattice engineering and interfacial charge regulation in high-efficiency SWIR QLEDs.

In parallel, the thick-shell strategy developed by Roshan et al. provided another effective route toward high-performance InAs QLEDs [61]. They synthesized InAs/ZnSe core/thick-shell QDs with approximately seven monolayers of ZnSe using amino-As precursors, achieving a PLQY of around 70% at 900 nm (Figure 8f). The thick ZnSe shell can spatially separate excitons from surface trap states, thereby suppressing non-radiative recombination and improving the thermal and environmental stability of the QDs during device operation. When these QDs were incorporated into an optimized device structure containing a PMMA interlayer to regulate electron injection, the resulting device achieved an EQE of 13.3% at 896 nm and a maximum radiance of 12 W·sr^−1^·m^−2^. This result indicates that thick-shell passivation and graded multi-shell lattice engineering are two complementary strategies for realizing high-efficiency InAs QLEDs: the former emphasizes physical isolation to reduce the influence of surface traps on excitons, whereas the latter focuses on suppressing interfacial defect formation through strain relaxation.

Recently, Li et al. achieved an important efficiency breakthrough in InAs QLEDs by introducing ZnF_2_ as a critical growth additive to synthesize InAs/InP/ZnSe/ZnS core/multi-shell QDs with near-unity PLQY at around 900 nm [64]. ZnF_2_ promotes uniform and defect-suppressed shell deposition while simultaneously passivating surface In vacancies, leading to highly monodisperse QDs. When these QDs were combined with a photo-crosslinked hole transport layer, TFB-CBPV, in devices, the improved charge balance and suppressed exciton quenching at the QD/HTL interface enabled the resulting QLEDs to achieve a record EQE of 20.5% at 905 nm (Figure 8h), a peak radiance of 581.4 W·sr^−1^·m^−2^, and an operational half-lifetime T_50_ of 550 h at an initial radiance of 50 W·sr^−1^·m^−2^. This represents the highest performance reported to date for Pb/Hg-free NIR QLEDs and marks a key step toward the practical application of InAs-based infrared light sources.

Despite these remarkable advances, most high-performance InAs QLEDs are still mainly limited to emission wavelengths below approximately 1100 nm. Extending InAs QLEDs into the deeper SWIR region remains challenging because long-wavelength emission requires larger InAs cores, which are typically accompanied by higher defect densities, broader size distributions, and lower PLQY. Recently, building on their previous work, Roshan et al. pushed this boundary by demonstrating InAs QD-LED electroluminescence beyond 1100 nm [62]. They employed an amino-As-based seeded growth method, using trioctylamine-alane as the reducing agent and ZnCl_2_ as an additive, to synthesize InAs cores with sizes ranging from 4.6 to 6.3 nm, followed by coating with 6–8 monolayers of ZnSe to form InAs/ZnSe core/shell QDs. In addition, to improve charge balance in these narrow-bandgap QDs, the device architecture was redesigned: Zn_0.9_Mg_0.1_O was used instead of ZnO as the ETL, and a double HTL composed of poly-TPD and PTAA was introduced. The optimized ITO/ZnMgO/QDs/poly-TPD/PTAA/MoOₓ/Au devices exhibited EL peaks at 1007, 1275, 1300, and 1410 nm, with corresponding EQEs of 6.20%, 3.75%, 2.04%, and 1.10%, respectively (Figure 8i,j). With increasing emission wavelength, both EQE and operational stability showed a systematic decline, mainly due to the reduced PLQY of large-sized QDs and enhanced trap-assisted non-radiative recombination. This work represents an important step toward RoHS-compliant InAs QD-LEDs covering the 1000–1400 nm SWIR window.

Looking forward, several challenges still need to be addressed before InAs QLEDs can move from laboratory demonstrations toward practical applications. First, extending emission further into the deeper SWIR region, especially beyond 1400 nm, while maintaining competitive EQE remains highly challenging, because larger InAs QDs usually possess higher densities of surface and interfacial defects. Second, although the operational lifetime of short-wavelength InAs QLEDs has been significantly improved, the stability of long-wavelength SWIR devices remains limited, requiring systematic investigation of degradation mechanisms under continuous high-current injection. Third, charge transport materials with better energy-level alignment need to be developed, because the band-edge positions of InAs QDs change significantly as the emission wavelength is tuned from the NIR to the SWIR region. Finally, scalable fabrication methods, such as inkjet printing and blade coating, are needed to overcome the limitations of small-area spin-coated devices while maintaining the precise multilayer architectures required for high efficiency. With continued progress in QD synthesis, shell engineering, surface passivation, interfacial design, and scalable processing technologies, RoHS-compliant InAs QLEDs are expected to become important infrared light sources for biosensing, machine vision, secure communication, and wearable optoelectronics.

### 3.3. Infrared Photodetectors

**Figure 9:**
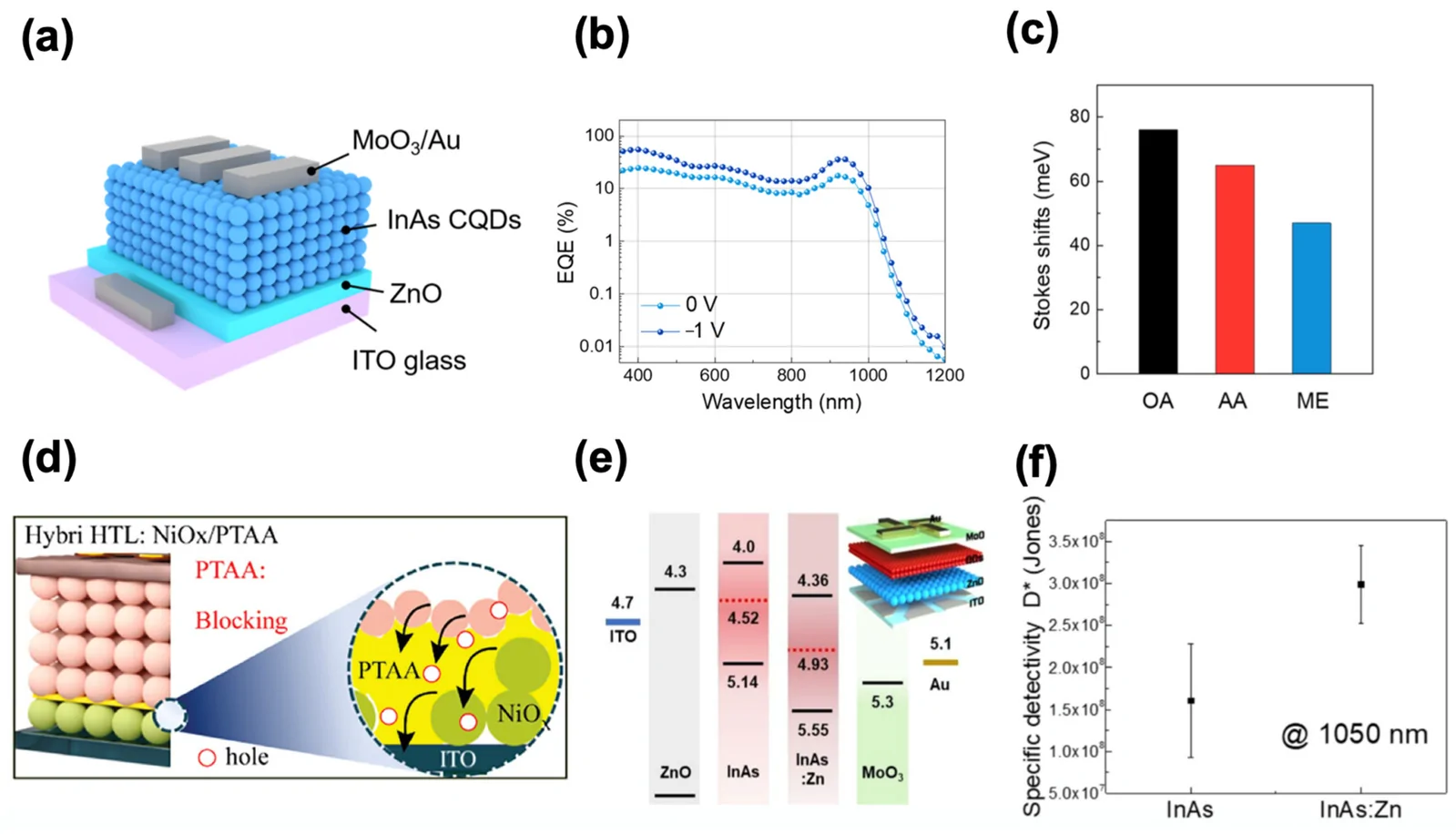
(**a**) Schematic of InAs photodiodes; (**b**) EQE of the device; (**c**) Stokes shifts in the device [65]; reprinted with permission from [65]. Copyright 2021, American Chemical Society. (**d**) A composite HTL of NiO_x_/PTAA of a p-i-n configuration [42]; reprinted with permission from [42]. Copyright 2023, The Authors; (**e**) band diagrams and structural schematics of the ITO/ZnO/QDs/MoO3/Au infrared photodiode; (**f**) detectivity at 1050 nm (1 mW/cm^2^, 1 kHz) [20]; reprinted with permission from [20]. Copyright 2023, The Authors.

InAs QDs have emerged as a transformative material for infrared photodetectors, particularly in the NIR to SWIR range. Their solution processability and size-tunable bandgap make InAs QDs promising candidates for infrared photodetection. However, achieving high responsivity, fast response speed, and low-noise operation generally requires effective surface passivation, controlled carrier transport, and optimized device interfaces. A significant challenge in utilizing InAs QDs has been the charge imbalance and trap states caused by unpassivated In and As dangling bonds, which can lead to Fermi-level pinning and reduced device performance. Choi et al. studied the stoichiometric balance of InAs QDs on their covalent surfaces through ligand exchange and applied them to high-performance photodetectors [65]. The study found that the synthesized InAs QDs had indium-rich surfaces with indium-carboxylate ligands (Z-type), leading to indium-rich non-stoichiometric surfaces and defect states. By using thiol-based ligands (such as 2-mercaptoethanol) for exchange, the In-carboxylate was successfully replaced with As-S covalent bonds (X-type ligands), achieving In/As stoichiometric balance and significantly reducing trap state density. Photodiodes based on this balanced InAs QD film (Figure 9a) achieved an EQE of 36% (Figure 9b) at 940 nm (−1 V bias). The Stokes shift decreased from 76 to 47 meV (Figure 9c) with a detectivity of 1.6 × 10^11^ Jones, and a response time as low as 65 ns, which is four times faster than PbS QD devices under the same conditions. This study provides a new approach for surface modulation of III-V QDs and the development of efficient optoelectronic devices.

In another study, Sun et al. addressed this issue with an innovative amphoteric ligand strategy using InBr_3_ in dimethylformamide, achieving a charge-neutral surface and enhanced mobility, resulting in photodiodes with a 30% EQE at 940 nm and a rapid response time of 2 ns [38]. Further advancements were made by Xia et al. in 2023, who developed a sequential co-passivation strategy combining X-type and Z-type ligands to improve the surface chemistry of InAs QDs [40]. This approach effectively reduced trap states while maintaining carrier transport, enabling photodetectors with an EQE of 37% at 950 nm and a specific detectivity of 1.9 × 10^11^ Jones, determined using experimentally measured noise at 10 kHz. In a subsequent study, Xia et al. investigated the operational degradation of InAs QD photodetectors and identified Zn ion migration from the ZnO ETL into the QD active layer as an important degradation mechanism [42]. To address this issue, they developed an inverted p-i-n architecture incorporating a C_60_ ETL and a hybrid NiO_X_/PTAA HTL (Figure 9d). The optimized devices achieved an EQE of 53% at 970 nm and a specific detectivity of 6.9 × 10^11^ Jones. Moreover, the devices retained 95% of their initial photocurrent after 19 h of continuously illuminated operation and over 80% of their initial performance after 80 days of storage. These findings demonstrate the importance of transport-layer engineering in suppressing ion-migration-induced degradation and improving device stability.

Beyond conventional surface ligand engineering, recent studies have further emphasized the importance of facet-specific defect control and internal electric-field management in improving InAs QD photodetectors. Xia et al. reported an improved facet and edge passivation strategy for near-infrared III–V colloidal quantum dot photodetectors, demonstrating that selectively addressing defect-rich regions at nanocrystal facets and edges can effectively suppress trap-assisted recombination and improve carrier extraction [66]. This work highlights that surface defects are not uniformly distributed across QD surfaces and that spatially controlled passivation provides an additional pathway for optimizing detector performance.

In addition to defect passivation, Park et al. demonstrated that internal field tailoring can simultaneously improve noise characteristics and carrier transport in colloidal quantum dot photodetectors operating beyond 1500 nm [67]. By engineering the internal electric field distribution within the device architecture, the authors achieved enhanced carrier separation and reduced noise, enabling high-speed infrared detection in the SWIR region. These advances indicate that future InAs QD photodetectors require integrated optimization of surface chemistry, nanoscale defect control, and device-level electric-field engineering to overcome the trade-off between responsivity, detectivity, and response speed.

Additionally, Kim et al. reported a new strategy for synthesizing infrared-active InAs QDs using zinc-based coordination compounds (ZnCl_2_-TOP) and applied them to high-performance infrared photodetectors [20]. The band diagrams and structural schematics of the ITO/ZnO/QDs/MoO3/Au infrared photodiode are shown in Figure 9e. The Zn additive chemically passivates the surface of InAs QDs during the co-reduction reaction, significantly narrowing the size distribution without the need for subsequent size selection. It also effectively removes surface oxidation defects, improving the PLQY (up to 1.26%) and colloidal stability. The Zn ions attached to the surface further modulate the energy levels and carrier concentration of InAs QDs, making their Fermi level more intrinsic, which is more suitable for constructing p-i-n structure photodiodes. Photodiodes based on InAs:Zn QDs exhibit excellent performance at a wavelength of 1050 nm: the dark current is reduced by two orders of magnitude, the response speed is faster, and the detectivity reaches 2.98 × 10^8^ Jones (Figure 9f), nearly doubling the performance compared to unmodified InAs devices. This study provides an effective surface and energy level engineering strategy for high-performance, Pb/Hg-free III-V QD infrared detectors.

Ban et al. studied the impact of different arsenic precursors ((TMSi)_3_As and (TMGe)_3_As) on the synthesis of InAs QDs and their photodetector performance [18]. The study found that QDs prepared with (TMGe)_3_As bind more oleic acid ligands on their surface, forming a thicker amorphous In-oleate shell layer, which leads to an increase in surface defects. Through InBr_3_ ligand exchange treatment, surface oxides and excess indium were effectively removed, achieving In/As stoichiometric balance and significantly improving surface quality. Ultimately, regardless of the precursor used, the surface-reconstructed InAs QDs exhibited similarly high performance in photodetectors: at a wavelength of 950 nm, the EQE reached 22–28%, the detectivity was about 4 × 10^11^ Jones, and the response time was 3.7 μs. This study demonstrates that precise surface engineering can eliminate the impact of synthesis pathway differences on device performance, providing a feasible strategy for high-performance, uniform InAs QD photodetectors. Shin et al. optimized the thickness of the InAs QD absorber layer, achieving a record EQE of 79% and a responsivity of 0.6 AW^−1^, with a detectivity of 3.1 × 10^11^ Jones [68]. Recently, Skorotetcky et al. introduced a hydrosilane reducing agent for InAs QDs, enhancing surface stability and optoelectronic quality, resulting in photodetectors with a 32% EQE at 900 nm and a faster response time of 4.4 μs [22].

The specific detectivity (D*) is an important figure of merit for evaluating the weak-light detection capability of infrared photodetectors. However, its reported value strongly depends on the noise evaluation method. The total noise generally includes shot noise associated with discrete carrier transport, thermal (Johnson-Nyquist) noise originating from thermal fluctuations, generation-recombination noise related to carrier trapping and recombination, and flicker (1/f) noise that becomes particularly significant at low frequencies. For devices in which the current noise spectral density is experimentally measured, D* can be expressed as

$$D_{\mathrm{meas}}^{*}\left(f\right)=\frac{R(f)\sqrt{A}}{i_{n,\mathrm{meas}}(f)}$$

where *R* is the responsivity, *A* is the active area, and *i_n_*_,*meas*_ is the experimentally measured current noise amplitude spectral density. When the noise is assumed to be dominated by dark-current shot noise, the detectivity is commonly estimated as:

$$D_{\mathrm{shot}}^{*}=\frac{R\sqrt{A}}{\sqrt{2qI_{\mathrm{dark}}}}$$

where *q* is the elementary charge and *I_dark_* is the dark current. However, this simplified expression assumes shot-noise-dominated behavior without additional noise enhancement and may not be applicable to photoconductors exhibiting substantial gain. Moreover, neglecting flicker noise, thermal noise, and trap-related fluctuations can lead to an overestimation of the actual detectivity. Therefore, experimentally determined and theoretically calculated D* values are distinguished in Table 4, and direct comparisons are made only with consideration of their respective measurement conditions.

Beyond noise evaluation, the performance of InAs QD photodetectors is governed by the interplay among dark current, responsivity, and response speed. Surface passivation and interface engineering can suppress defect-assisted leakage and dark current, thereby reducing noise and potentially improving detectivity. However, excessive interfacial barriers or strongly insulating ligands may impede photocarrier extraction, resulting in reduced responsivity and slower carrier transport. Conversely, trap-assisted photoconductive gain can enhance responsivity by extending the effective carrier lifetime, but often compromises response speed and may introduce additional low-frequency noise. Therefore, achieving high detectivity does not necessarily guarantee fast operation. Future optimization of InAs QD photodetectors should focus on simultaneously minimizing dark current and trap-related noise while preserving efficient charge extraction and rapid temporal response.

The development of wearable health monitoring technologies imposes additional requirements on infrared photodetectors, including mechanical flexibility, low power consumption, and reliable long-term operation. As a representative demonstration using an alternative Pb- and Hg-free QD platform, Li et al. developed ultrathin, self-powered Cu-In-Se QD photodetectors for wearable photoplethysmography monitoring [74]. The devices employed an approximately 40 nm-thick QD absorbing layer and achieved a specific detectivity of 2.10 × 10^12^ Jones at zero bias, with a spectral response extending from 250 to 1050 nm. Their successful integration into wearable photoplethysmography systems highlights the importance of combining suitable QD absorbers with interface engineering and mechanically compliant device architectures. Although this demonstration employed Cu-In-Se rather than InAs QDs, it provides a relevant reference for the future development of flexible, self-powered InAs-based photodetectors.

These advancements underscore the potential of InAs QDs in developing high-performance, stable, and Pb/Hg-free infrared photodetectors, paving the way for their widespread application in next-generation optoelectronic devices. The strategic engineering of surface passivation, device architecture, and synthesis methods continues to enhance the capabilities of InAs QDs, positioning them as a key material in the field of infrared detection.

### 3.4. Field-Effect Transistors (FETs)

**Figure 10:**
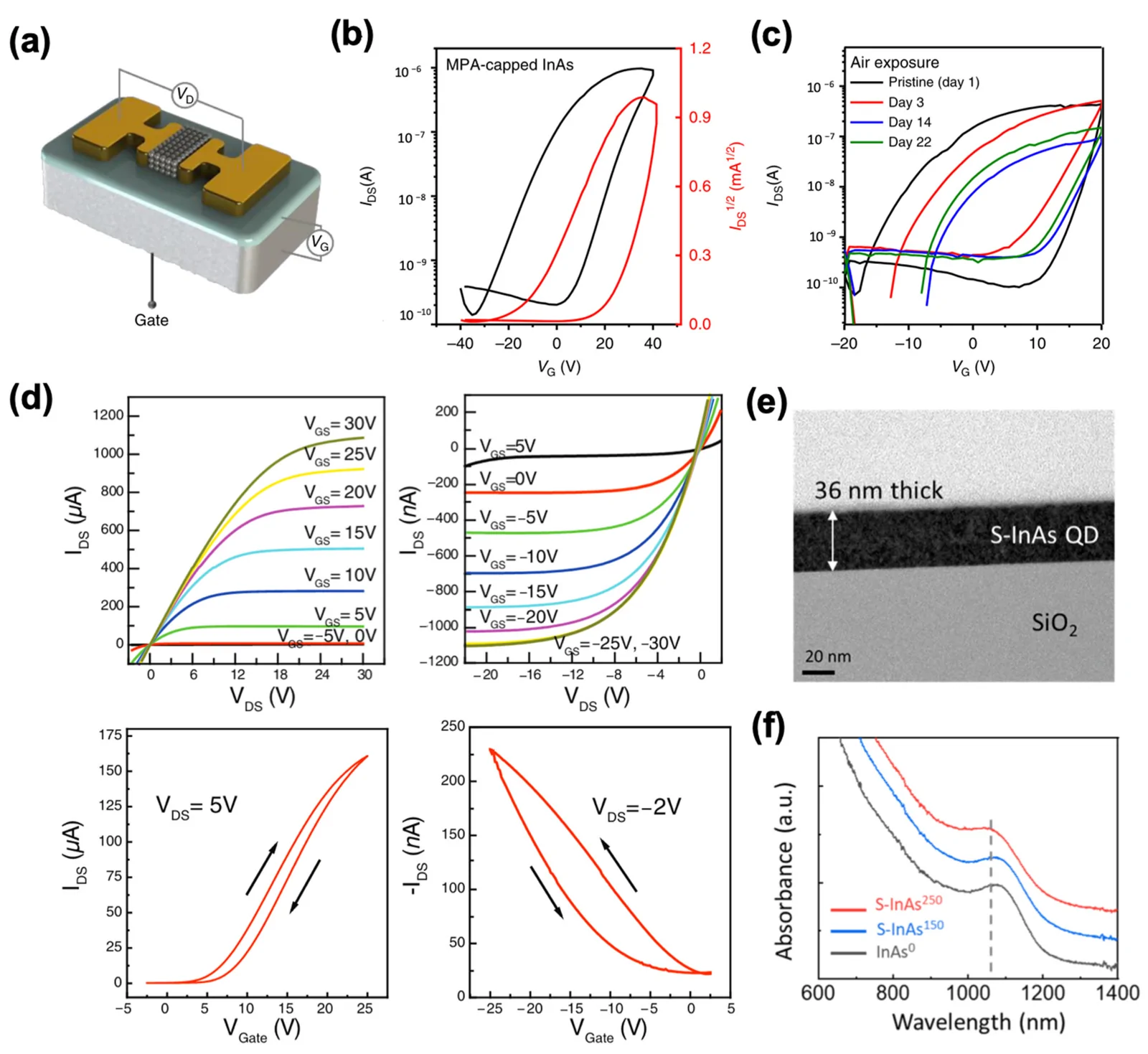
(**a**) Device configuration of the InAs QD FET; (**b**) transfer curves of MPA-treated InAs QD FETs; (**c**) transfer curves of FETs based on MPA-treated InAs QDs after ambient air storage for different durations [17]; reprinted with permission from [17]. Copyright 2018, The Authors. (**d**) FET performance of intrinsic versus Cd-doped InAs NCs. Left: output and transfer characteristics of intrinsic InAs NCs exhibit n-type conductivity; right: output and transfer curves of Cd-doped InAs NCs, demonstrating effective doping and p-type conduction [75]; reprinted with permission from [75]. Copyright 2020, Wiley-VCH GmbH. (**e**) TEM micrograph of sulfide-vapor-processed InAs QD film; (**f**) UV-Vis absorption spectra of InAs^0^, S-InAs^150^, and S-InAs^250^ films [39]; reprinted with permission from [39]. Copyright 2021, The Authors.

FETs are fundamental components in modern electronics for controlling signals. InAs QDs have attracted interest as semiconductor channels in FETs owing to their tunable electronic structures and potential for solution-processed thin-film fabrication. However, their electrical performance is strongly influenced by surface defects, ligand chemistry, doping, and interparticle coupling. InAs QDs and nanocrystals have been explored extensively for their ability to serve as semiconducting channels in FETs, offering tunable electronic characteristics through various surface modifications and doping strategies. Song et al. demonstrated the use of InAs QD films in FETs (Figure 10a), achieving remarkable n-type characteristics with electron mobilities up to 2.5 × 10^−3^ cm^2^ V^−1^ s^−1^ through ligand exchange processes involving Br^−^ and MPA (Figure 10b). This approach not only enhanced carrier mobility by reducing trap state density but also ensured air stability over 22 days (Figure 10c), highlighting the potential of InAs QDs for flexible electronics and optoelectronic integration [17].

Further advancements were made by Asor et al., who successfully converted initially n-type InAs NCs into p-type semiconductors through post-synthetic Cd doping. This transformation enabled the construction of high-performance FET devices with stable hole conduction characteristics, achieving hole mobilities of 1.5 × 10^−3^ cm^2^/V·s, as shown in Figure 10d [75]. The Cd atoms replaced near-surface In atoms, serving both as p-type dopants and forming a protective layer that significantly enhanced device stability in air, maintaining performance for over 300 days. This development is crucial for the realization of printed electronics based on NC-based p-n homojunction devices.

Beyond solution-phase ligand exchange, vapor-phase chalcogenation has proven especially effective. Koh et al. explored the impact of hydrogen sulfide (H_2_S) vapor treatment on InAs QDs for FET applications, achieving surface sulfuration that improved air stability and charge transport performance [39]. Figure 10e shows a TEM micrograph of a sulfide-vapor-processed InAs QD film which exhibited enhanced n-type conduction with electron mobilities reaching 1.7 × 10^−3^ cm^2^ Vs^−1^ and demonstrated significant photoconductive responses under NIR illumination, with photocurrent behavior modulated by gate voltage. This vapor treatment offers a potential route to improved charge transport and stability in InAs QD-based optoelectronic devices.

Overall, the versatility of InAs in FET applications is underscored by its ability to undergo various modifications that tailor its electronic properties, enabling the development of both n-type and p-type devices. These advancements pave the way for InAs-based FETs in complementary logic circuits, as demonstrated by Yoon et al., who synthesized p- and n-type InAs QDs for use in NOT, NOR, and NAND logic gates. The comparable mobilities of holes and electrons in these devices facilitate the construction of robust complementary logic circuits, showcasing the potential of InAs QDs in next-generation electronic applications.

### 3.5. Cross-Device Comparison: Interfacial Loss Mechanisms and Design Trade-Offs

Although interface engineering is essential for InAs QD optoelectronics, its optimal implementation depends strongly on the operating requirements of different devices. Solar cells and photodetectors require efficient photocarrier separation and extraction, whereas QLEDs favor radiative recombination and balanced carrier injection. FETs, in contrast, rely on gate-controlled charge transport and stable carrier polarity. Consequently, similar interfacial modifications may produce different functional benefits and performance trade-offs across these device platforms.

Surface passivation and interparticle electronic coupling provide a representative example. Song et al. demonstrated that ligand reconstruction using Br^−^ or MPA improved charge transport in InAs QD FETs while also enabling efficient charge separation and extraction in InAs/PbS heterojunction solar cells [17]. Similarly, thiol- and InBr_3_-based surface treatments reduced trap-related losses and improved photocarrier collection in infrared photodetectors [38,65]. However, enhanced electronic coupling does not necessarily guarantee improved device performance, because changes in surface chemistry can also modify carrier concentration, energy-level alignment, and leakage pathways. Thus, ligand engineering must balance defect passivation and transport according to the intended device function.

A second important distinction concerns carrier confinement and interfacial charge transfer. In InAs QLEDs, strain-managed core/shell structures and suitable interlayers suppress nonradiative recombination and interfacial exciton quenching. For example, Zhao et al. combined multi-shell passivation with PVK interlayer engineering to improve charge balance and electroluminescence efficiency [33]. By contrast, photodetectors and solar cells require photogenerated carriers to reach selective contacts efficiently. Therefore, shell composition, thickness, and band offsets must balance defect suppression against carrier-transfer requirements. Stronger confinement is not necessarily beneficial for every device architecture.

Transport-layer interfaces and functional-layer thickness introduce further device-specific trade-offs. In QLEDs, interlayers can improve charge balance by restricting excessive carrier injection [33], whereas selective contacts in solar cells and photodetectors must promote carrier extraction while suppressing recombination and leakage. For example, InAs QD ETL improved charge extraction and photostability in organic solar cells [51], while inverted photodiode architectures mitigated transport-layer-related degradation [42]. Similarly, thicker photoabsorbing layers can enhance infrared absorption but may introduce additional collection losses, whereas excessive thickness in charge-selective interlayers can increase transport resistance or reduce effective photocurrent [51,52,68].

These comparisons reveal that the optimal interface is determined by the dominant loss mechanism and carrier dynamics of the target device, rather than by maximizing an individual material property such as PLQY or carrier mobility. As summarized in Table 5, improved InAs QD device performance requires coordinated optimization of surface passivation, electronic coupling, energy-level alignment, and interfacial stability. Importantly, the proposed trade-offs represent mechanistically motivated design considerations; quantitative relationships should be established through controlled experiments and comparable device measurements. This device-oriented perspective provides a common framework for interpreting interface-engineering strategies while identifying the distinct requirements of different InAs QD optoelectronic applications.

## 4. Conclusions and Emerging Opportunities

The progress of InAs QD optoelectronics demonstrates that advances in nanocrystal synthesis and optical quality represent necessary but insufficient conditions for realizing efficient, stable, and scalable devices. The central insight emerging from the present review is that the functional performance of InAs QDs is determined by a hierarchy of interfaces extending from the nanocrystal core/shell boundary to ligand-mediated interparticle contacts, charge-transport heterojunctions, and processing-dependent device interfaces.

These interfaces regulate different but strongly coupled physical processes. Core/shell engineering determines carrier confinement, strain accommodation, and defect-related recombination, whereas surface reconstruction and ligand exchange influence surface stoichiometry, energetic disorder, and interparticle electronic coupling. At the device level, transport-layer and contact engineering further determine charge injection and extraction, recombination pathways, leakage currents, and operational degradation.

Importantly, the relative importance of these mechanisms varies across applications. QLEDs require effective radiative recombination together with balanced charge injection, while photodetectors and photovoltaic devices demand efficient carrier extraction, suppressed parasitic currents, and reduced nonradiative losses. FETs provide complementary insight into the transport and doping characteristics of electronically coupled QD films. Notably, advances in core/shell passivation and charge-transport interface engineering have enabled InAs QLEDs to achieve a maximum EQE of 20.5% at an emission wavelength of 905 nm, representing a benchmark for NIR electroluminescence. Consequently, no single interfacial modification can be assumed to optimize all InAs QD devices equally.

Future research should therefore move beyond the independent improvement of nanocrystal optical quality and individual device-performance metrics toward coordinated optimization across different interface levels. Particular priorities include establishing quantitative correlations between interfacial structure and carrier-loss mechanisms, developing passivation strategies compatible with efficient electronic transport, identifying application-specific energy-level and contact requirements, and preserving interfacial quality during large-area film formation and device operation.

Equally important is the development of consistent characterization and reporting practices that distinguish the fundamental electronic and optical properties of InAs cores from improvements achieved through shell growth, surface passivation, and device-level interface engineering. The combination of advanced surface analysis, carrier-dynamics measurements, electrical characterization, and controlled operational-stability tests will be essential for establishing reliable structure-interface-performance relationships. Such an approach can guide the rational development of InAs QD technologies in which synthetic chemistry, multilevel interface engineering, and device-performance optimization are treated as interconnected design problems rather than separate stages of optimization.

To bridge the gap between fundamental nanomaterial science and real-world optoelectronic applications, we propose that the following seven emerging research opportunities merit particular attention:

(1) Data-Driven and Sustainable Synthesis: Transitioning toward sustainable “green chemistry” protocols that utilize low-toxicity precursors and benign solvents. Crucially, integrating automated flow-reactor technologies with machine learning algorithms and high-throughput screening will be essential for rationally navigating the vast synthetic parameter space, thereby ensuring precise size control and industrial-scale reproducibility.

(2) Deciphering and Engineering Interfaces: Advancing toward a deterministic, atomic-level understanding of QD surfaces. This requires the synergistic combination of advanced operando characterization techniques and multiscale computational modeling (e.g., Density Functional Theory) to design robust, defect-tolerant core/shell architectures and tailored inorganic ligands that inherently suppress Auger recombination and surface trapping.

(3) Pioneering Hybrid Architectures and Metamaterials: Moving beyond isolated QD systems by rationally integrating InAs QDs with 2D materials, plasmonic arrays, or topological photonic microcavities. Such hybrid platforms are critical for manipulating light-matter interactions at the nanoscale, unlocking exotic optoelectronic phenomena, and enabling multifunctional devices such as room-temperature single-photon emitters and ultra-broadband photodetectors.

(4) Engineering Energy-Aligned Charge Transport Layers: Developing novel electron and hole transport materials specifically tailored to match the narrow bandgaps and deep energy levels of infrared-active InAs QDs. Conventional wide-bandgap transport layers often introduce severe energy barriers at the QD interface, impeding efficient charge extraction or injection. Future device engineering must prioritize the design of new organic, inorganic, or hybrid charge transport layers with synthetically tunable work functions and high carrier mobilities to minimize interfacial voltage losses and maximize device-specific detectivity.

(5) Scalable Fabrication of Large-Area Devices: Transitioning from lab-scale, small-area pixels to large-area optoelectronic devices, encompassing both broad-area infrared emitters and large-active-area single-element photodetectors, presents substantial engineering hurdles. Conventional deposition methods like spin-coating often fail to maintain film uniformity and suppress pinhole formation over macroscopic dimensions. Future advancements must prioritize scalable, large-area coating technologies, such as slot-die coating, blade coating, and inkjet printing, to deposit homogeneous, densely packed InAs QD thin films.

(6) Developing Tandem and Stacked Device Architectures: To further push the efficiency limits and operational stability of InAs QD-based optoelectronics, exploring tandem or vertically stacked architectures is a critical next step. By integrating InAs QD active layers with other tailored bandgap materials (such as perovskites, silicon, or wide-bandgap QDs), researchers can achieve ultra-broadband multispectral detection or significantly boost the external quantum efficiency of light-emitting diodes. Realizing high-performance tandem structures necessitates intricate engineering of intermediate recombination layers (e.g., tunnel junctions) to ensure efficient carrier transport, current matching, and optical transparency. Crucially, these stacked configurations not only maximize photon harvesting and emission but also effectively distribute thermal and electrical stress, thereby substantially extending the long-term operational lifespan of the devices.

(7) Exploring Scalable Manufacturing and Imaging-Compatible Platforms: Reproducible film deposition, high-fidelity patterning, and process compatibility are important for translating promising discrete-device results into practical formats. Initial demonstrations involving silicon readout electronics motivate further evaluation of CMOS-compatible processing, array-level uniformity, device yield, pixel-to-pixel variation, and encapsulation. Wafer-scale production, high-resolution focal-plane arrays, and silicon-photonics interfaces remain longer-term opportunities that require systematic experimental validation rather than being capabilities that can be assumed from individual device demonstrations.

With sustained interdisciplinary efforts spanning synthetic chemistry, interfacial characterization, and device engineering, InAs QDs may provide new routes toward infrared optoelectronics with reduced reliance on Pb- and Hg-containing materials. Progress in interface engineering, reproducible device performance, and scalable processing could broaden opportunities in biomedical imaging, machine vision, optical communications, and environmental sensing, provided that full device composition, operational stability, and practical manufacturability are carefully assessed.

## Figures and Tables

**Table 1:** Summary of representative synthesis methods, reaction conditions, and optical properties of InAs QDs.

Methods	Arsenide Source	Indium Source	Reducing Agent/Ligand/Solvent	Reaction Temperature (°C)	QD Size (nm)	Exciton/Emission Range (nm)	HWHM (nm/meV)	FWHM (nm/meV)	P/V Ratio	PLQY (%)	Refs
Hot injection	(NMe_2_)_3_As	InCl_3_	P(Net_2_)_3_, OAm	190	3.4 ± 0.5	Abs: 720–800	75–80 meV *	/	1.0–1.3 *	7–20	[6]
	(NMe_2_)_3_As	InCl_3_	DMEA-AlH_3_, OAm, ZnCl_2_	240	2.8 ± 0.2	Abs: 766 Em: 837	114 meV *	100 meV	1.3 *	42	[23]
	(NMe_2_)_3_As	InCl_3_	P(Net_2_)_3_, OAm	190	7.4 ± 0.2	Abs: 1600 Em: 1030	52 nm *	111 nm*	1.02 *	12	[24]
	(NMe_2_)_3_As	InCl_3_	ZnCl_2_, OAm	170	3.4 ± 0.5	Abs: 850–1550	65–135 nm *	/	1.1–1.13 *	/	[22]
One-pot approach	(NMe_2_)_3_As	InCl_3_	DIBAL-H, OAm	240	6.2 ± 0.68	Abs: 750–1450	85–173 meV *	/	1.0–1.1	/	[9]
Continuous injection	(TMS)_3_As	In(OAc)_3_	ODE, OA	300	6.1 ± 0.9	Abs: 1600	155 nm	/	1.0 *	/	[11]
	(TMSi)_3_As, (TMGe)_3_As	In(OAc)_3_	ODE, DOA	300	4.0 ± 0.2	Abs: 825–950 Em: 820–940	73–140 nm *	65–75 nm	1.5–2.8	0.5	[18]
Cluster precursor synthesis method	(TMS)_3_As	In(OAc)_3_	TOP, OAm, ODE	270	1.8 ± 0.55	Abs: 937–1110	50–61 meV *	/	1.6–1.8 *	/	[14]

* Result estimated on the basis of the original literature data.

**Table 2:** Performance comparison of InAs QD-based solar cells and other quantum dot photovoltaic devices.

QD Type	Voc (V)	PCE (%)	FF (%)	J_SC_ (mA/cm^2^)	Stability (h)	Refs.
InAs-MPA	0.54	7.92	64.80	22.30	312	[17]
InAs-SL/nAs-MLs	0.22/0.42	0.85/2.4	40.5/46.9	9.5/12.1	/	[52]
InAs/GaAs	0.90	18.70	80.00	26.00	/	[53]
InAs/GaAs_0.82_Sb_0.18_	0.85	17.31	72.37	28.19	/	[54]
InAs/GaAs	0.84	16.80	76.30	26.00	/	[55]
PbSe	0.64	11.6	66.3	28.11	1440	[56]
PbS	0.7	13.3	67.35	28.22	700	[57]
AgBiS_2_	0.49	9.1	68.1	27.1	3	[58]
CsPbI_3_	1.26	16.64	74.8	17.65	200	[59]

**Table 3:** Performance comparison of InAs QD-based QLEDs.

Shell Structure	λ_EL_ (nm)	EQE (%)	PLQY (%)	Device Structure	Max Radiance (W sr^−1^ m^−2^)	T_50_ (h)	Refs.
InAs/ZnSe	947	5.5	≈42 ± 4 (solution)	ITO/PETDOT:PSS/ Poly-TPD/QD/TPBi/LiF/Al	0.15	32	[23]
In(Zn)As/ In(Zn)P/GaP/ ZnS	1006	13.3	73 (solution)	ITO/ZnO/PVK/ PEIE/CQDs/Poly-TPD/MoO_3_/Ag	2.2	/	[33]
In(Zn)As/ In(Zn)P/GaP/ ZnS	857	4.6	75 (solution)	ITO/ZnO/PEIE/QD/Poly-TPD/MoO_3_/Al	8.2	/	[34]
InAs/InP/ ZnSe/ZnS	905	20.5	≈100 (solution)	ITO/PETDOT:PSS/ TFB:CBPV/QDs/ZMO/Al	581.4	550	[36]
InAs/ZnSe (thick shell)	896	13.3	55	ITO/ZnO/PMMA/QD/Poly-TPD/MoO_3_/Al	12	0.55 (unprotected)/ 0.8 (protected)	[61]
InAs/ZnSe (QD1)	1007	6.20	45 ± 5 (sol.) 25 ± 3 (film)	ITO/ZnMgO/QDs/poly-TPD/PTAA/ MoO_x_/Au	2.3	/	[62]
InAs/ZnSe (QD2)	1275	3.75	35 ± 4 (sol.) 12 ± 1 (film)	ITO/ZnMgO/QDs/poly-TPD/PTAA/ MoO_x_ /Au	2.3	/	[62]
InAs/ZnSe (QD3)	1300	2.04	30 ± 3 (sol.) 10 ± 1 (film)	ITO/ZnMgO/QDs/poly-TPD/PTAA/ MoO_x_/Au	1.9	/	[62]
InAs/ZnSe (QD4)	1410	1.10	17 ± 2 (sol.) 9 ± 1 (film)	ITO/ZnMgO/QDs/poly-TPD/PTAA/ MoO_x_ /Au	1.0	/	[62]

**Table 4:** Performance comparison of InAs colloidal quantum dots and other quantum dot infrared photodetectors.

Type of QDs	Wavelength (nm)	EQE (%)	Dark J (A cm^−2^)	R (A W^−1^)	D* (Jones)	Noise-Evaluation Method	Response Time (ns)	Ref.
InAs	935	18 (0 V); 36 (−1 V)	/	/	1.6 × 10^11^	Measured PSD (1 kHz; 0 V)	65 (fall; 0 V)	[65]
	940	30 (0 V)	≈7 × 10^−7^ (−1 V)	0.22	1.0 × 10^11^	Measured PSD (500 kHz)	2 (fall; 0 V)	[38]
	950	37 (0 V)	/	/	1.9 × 10^11^	Measured PSD (10 kHz)	1000 (rise); 1400 (fall; 0 V)	[40]
	940	22 (0 V); 28 (−1 V)	≈2 ×10^−7^ (−1 V)	/	≈4 × 10^11^	Calculated	3700 (fall; 0 V)	[18]
	970	53	/	0.40	6.9 × 10^11^	Measured PSD (10 kHz; 0 V)	/	[42]
	940	79 (optimized bias)	/	0.60	3.1 × 10^11^	Calculated	46 (fall; −1 V)	[68]
	1140	75 ± 3 (−0.5 V)	≈1 × 10^−7^ (−0.5 V)	/	/	/	10 (reported)	[66]
	1500	≈22 (−0.15 V)	9.7 × 10^−7^ (−0.2 V)	/	5.2 × 10^11^	Shot-noise Calculated	24 (rise; −0.15 V)	[67]
PbS	1310	70.8 (0 V)	2.74 × 10^−7^ (−0.5 V)	0.765	3.57 × 10^11^	Calculated	910 (rise);	[69]
PbSe	1460	23.96 (−0.5 V)	1.49 × 10^−4^ (−0.5 V)	0.282	4.08 × 10^10^	Calculated	12,000 (rise);	[70]
HgTe	/	/	/	<10^−2^	1.0 ×10^10^	Calculated	/	[71]
InSb	1200	75 (−1 V)	1.48 ×10^−4^ (−1 V)	0.7	1.02 ×10^11^	Shot-noise Calculated	5400 (fall; −1 V)	[72]
InAs/GaSb T2SL	/	26.7 (−0.1 V)	2.3 ( −0.1 V)	1.2	1.3 ×10^9^	Calculated	/	[73]

PSD = experimentally measured current noise spectral density.

**Table 5:** Device-specific interfacial loss mechanisms, engineering strategies, and performance trade-offs in InAs QD optoelectronics.

Device	Critical Interface	Dominant Loss Mechanism	Engineering Strategy	Performance Trade-Off
Solar cells	QD/QD junction; QD/transport layer	Interfacial recombination; extraction barriers	Ligand reconstruction; selective contacts	Passivation vs. carrier extraction
QLEDs	Core/shell; QD/ETL and QD/HTL	Nonradiative recombination; exciton quenching; carrier imbalance	Multi-shell passivation; interlayer engineering	Carrier confinement vs. injection
Photodetectors	QD surface; inter-QD; transport-layer interfaces	Trapping; dark current; noise; collection losses	Surface reconstruction; selective contacts; thickness optimization	Electronic coupling vs. leakage; absorption vs. extraction
FETs	Inter-QD; QD/dielectric; source/drain contacts	Trap-limited transport; contact resistance; unstable carrier polarity	Ligand exchange; sulfide treatment; doping	Mobility vs. switching control; doping vs. stability

## Data Availability

No new data were created or analyzed in this study. Data sharing is not applicable to this article.
